# Recent Advances in MoS_2_-Based Saturable Absorbers for Mode-Locked Fiber Lasers

**DOI:** 10.3390/nano16150911

**Published:** 2026-07-24

**Authors:** Jiahao Huang, Jiancheng Zheng, Xin Xiong, Yuxian Yang, Xiyan Huang, Chibiao Liu

**Affiliations:** Fujian Key Lab of Agriculture IOT Application, School of Information Engineering, Sanming University, Sanming 365004, China; 20240865218@fjsmu.edu.cn (J.H.); 20240865149@fjsmu.edu.cn (X.X.); yangyx@fjsmu.edu.cn (Y.Y.); xyhuang@fjsmu.edu.cn (X.H.)

**Keywords:** molybdenum disulfide, saturable absorber, mode-locked fiber laser, nonlinear optics, ultrashort pulse, nonlinear optical properties

## Abstract

Ultrashort pulse mode-locked fiber lasers demonstrate significant application potential in optical communication, precision measurement, and ultrafast photonics. As the critical component for pulse generation, the performance of saturable absorbers directly dictates the output characteristics of these lasers. To address the limitations of traditional saturable absorbers regarding operating bandwidth, fabrication cost, and environmental stability, two-dimensional transition metal dichalcogenides represented by molybdenum disulfide have emerged as research hotspots in the field of novel saturable absorbers due to their broadband tunability, superior nonlinear optical response, and flexible fabrication processes. This review focuses on molybdenum disulfide saturable absorbers and systematically summarizes their nonlinear optical properties, mainstream fabrication strategies, and recent application progress in mode-locked fiber lasers. Furthermore, the advantages and challenges concerning key performance metrics such as pulse stability, output power, and pulse duration are comprehensively evaluated. Finally, future perspectives on critical issues including fabrication process optimization, long-term stability enhancement, and composite structure design are discussed. This work aims to provide theoretical references and technical support for the practical application of high-performance ultrashort pulse fiber lasers.

## 1. Introduction

Ultrafast fiber lasers have emerged as key core light sources in optical communications, precision micro–nano fabrication, biomedical imaging, and nonlinear spectroscopy, driven by their superior beam quality, efficient thermal management, and compact all-fiber architecture. Among the techniques for generating ultrashort pulses, passive mode-locking is widely recognized as a robust method for achieving high-energy ultrashort pulses. This is primarily attributed to its distinct advantages, including the elimination of external modulation, simplified system architecture, and high operational stability. As the core functional component in passive mode-locking technology, the nonlinear optical properties of the saturable absorber directly determine the mode-locking threshold, pulse stability, output pulse duration, and wavelength tuning range of the laser [1].

Although traditional semiconductor saturable absorber mirrors (SESAMs) have achieved commercial viability, their deployment in next-generation high-power fiber lasers is fundamentally constrained. State-of-the-art ultrafast fiber oscillators now routinely deliver average powers exceeding 10 W with pulse energies > 100 nJ, whereas conventional SESAMs typically exhibit irreversible thermal degradation at average intracavity powers above ~1–2 W. Furthermore, SESAMs are limited by narrow operational bandwidths (<100 nm) and costly epitaxial growth processes that hinder wavelength tunability and scalable integration [2,3]. These quantitative bottlenecks have motivated the urgent search for alternative saturable absorbers capable of meeting the demands of modern high-power, broadband ultrafast optics.

In this context, two-dimensional layered materials have emerged as a promising platform for next-generation saturable absorbers. Their atomic-level thickness, large specific surface area, and unique quantum confinement effects yield distinct nonlinear optical responses and ultrafast carrier dynamics [4,5,6,7]. Among transition metal dichalcogenides, molybdenum disulfide (MoS_2_) has been widely investigated as a representative candidate. Exhibiting tunable saturable absorption across the near-infrared to mid-infrared spectral ranges, strong excitonic interactions, and favorable chemical stability, MoS_2_ has attracted significant attention as a post-graphene saturable absorber, offering broadband operation from the near-infrared to mid-infrared ranges, inherent environmental stability, and layer-dependent tunable nonlinearity [8,9,10,11,12]. Relative to single-element carbon-based materials such as graphene and black phosphorus (BP), MoS_2_ exhibits enhanced environmental stability and fabrication reproducibility, while enabling parameter optimization—including modulation depth and saturation intensity—through layer-number control and heterostructure design [13,14,15,16,17,18]. Table 1 demonstrates the key performance parameters of representative saturable absorbers for mode-locked fiber lasers.

This review addresses critical gaps in the current understanding of MoS_2_-based saturable absorbers for mode-locked fiber lasers. While previous reviews have established foundational knowledge on basic properties and early-stage applications, this work extends the discussion by linking material structure to ultrafast carrier dynamics through mechanistic analysis of representative heterostructure and composite designs. We further evaluate performance trade-offs across spectral bands and operational conditions, explicitly discussing intrinsic limitations such as interface-induced damage thresholds and environmental sensitivity in hybrid systems. Finally, we outline realistic pathways toward industrial deployment, grounded in current manufacturing capabilities and reliability requirements, aiming to provide actionable guidance for designing next-generation ultrafast lasers beyond laboratory-scale demonstrations.

## 2. Properties and Fabrication

### 2.1. Optical and Nonlinear Properties

As a representative transition metal dichalcogenide, molybdenum disulfide has emerged as a prominent candidate for SAs in mode-locked ultrafast fiber lasers, primarily due to its distinctive layered crystal structure and unique optical characteristics. The application potential of this material in mode-locking technology is fundamentally rooted in its controllable structural properties, notable nonlinear optical responses, broad spectral adaptability, and tailorable stability.

The nonlinear optical characteristics serve as the foundation for MoS_2_ functioning as a mode-locked SA, with the saturable absorption effect originating predominantly from carrier saturation mechanisms. When the incident optical intensity falls below the saturation threshold, valence band electrons in MoS_2_ are excited to the conduction band, resulting in carrier absorption and consequently low transmittance. Conversely, as the incident intensity reaches the saturation threshold, extensive excitation of valence band electrons occurs, leading to absorption saturation and a significant enhancement in transmittance [19,35]. This dynamic process facilitates pulse selection and shaping, thereby enabling the mode-locking operation [36,37,38,39].

**Figure 1:**
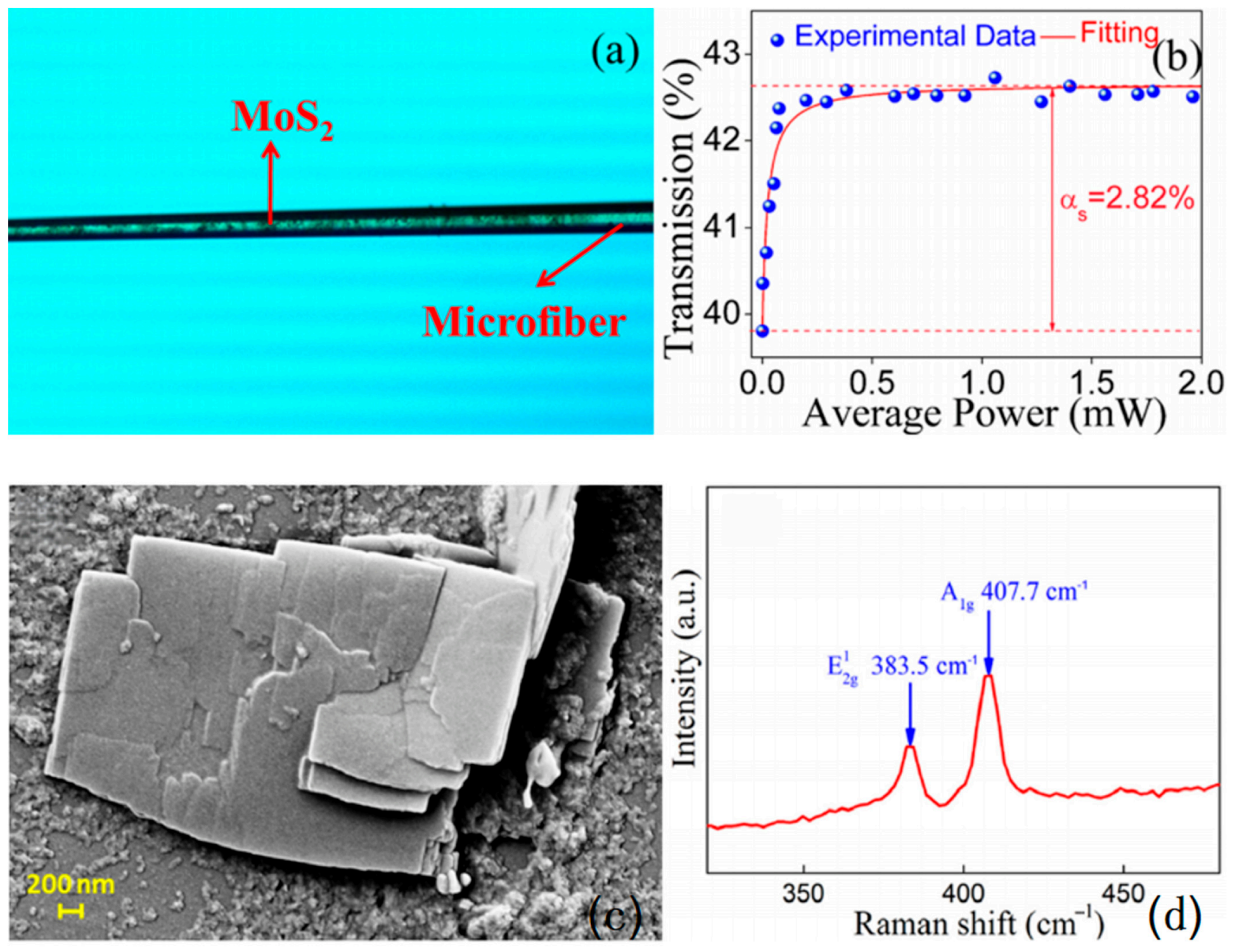
Characterization of few-layer MoS_2_ SA and its nonlinear optical properties. (**a**) Microscopy image of the microfiber-based MoS_2_ SA, showing the uniform deposition of MoS_2_ nanosheets on the microfiber surface; (**b**) Measured saturable absorption curve and the corresponding fitting curve, indicating a modulation depth (α_s_) of ~2.82% and non-saturable loss of ~57.34%; (**c**) Scanning electron microscopy image of few-layer MoS_2_ nanosheets, revealing their typical layered structure with a lateral size of several hundred nanometers (scale bar: 200 nm); (**d**) Raman spectrum of few-layer MoS_2_, showing the characteristic E^1^_2_g and A_1_g vibrational modes at 383.5 cm^−1^ and 407.7 cm^−1^, respectively, confirming the few-layer nature of the synthesized material [9]. Reprinted from Ref. [9].

Molybdenum disulfide features a layered van der Waals structure, with covalently bonded S-Mo-S monolayers connected by weak interlayer forces. This architecture determines a strong layer-dependent optical performance. As the layer number increases, the band structure transitions from a direct to an indirect bandgap, significantly altering optical absorption and nonlinear parameters. Few-layer MoS_2_ has been widely used for mode-locked SAs, as it combines the strong nonlinear response characteristic of monolayers with improved structural stability compared to single-layer films. The exact optimal layer number depends on specific application requirements, including pump wavelength and cavity configuration, and varies across different experimental realizations. Precise layer control is therefore essential for optimizing mode-locking performance [9]. However, optical performance metrics such as modulation depth and non-saturable loss are heavily dependent on the integration scheme. For instance, the microfiber-based device shown in Figure 1 exhibits a modulation depth of ~2.82% but suffers from a high non-saturable loss of ~57.34%. In a practical all-fiber ring cavity, such significant non-saturable loss acts as a substantial intracavity insertion loss. This not only raises the laser oscillation threshold—requiring higher pump power to initiate mode-locking—but also inevitably reduces the slope efficiency of the laser output. In comparison to other integration schemes that will be discussed later, such as end-face or D-shaped fiber configurations, microfiber devices often encounter greater challenges in suppressing this parasitic loss. This is largely attributed to the difficulties in controlling the evanescent field interaction length and ensuring material deposition uniformity along the tapered region. Therefore, balancing the modulation depth with low non-saturable loss remains a critical optimization target for different device geometries. Unlike traditional alternatives, MoS_2_ exhibits a broadband response, achieving saturable absorption across 1 μm, 1.55 μm, and 2 μm bands. This versatility covers the primary operational wavelengths of ultrafast fiber lasers without requiring complex structural modifications, providing a practical foundation for multi-wavelength mode-locking systems [40,41,42,43]. Long-term stability is critical but often degraded under environmental factors such as high temperature and humidity, which induce oxidation and interlayer exfoliation. To mitigate this, encapsulation and composite structuring have been employed. Polymer encapsulation, for instance, effectively isolates moisture and oxygen, ensuring stable modulation over extended operation [44,45]. Comparative studies have shown that monolayer MoS_2_ devices can achieve mode-locking thresholds as low as −200 µW in similar 1.55 µm cavity configurations [9], but they typically exhibit rapid degradation under continuous operation due to environmental oxidation within hours. Conversely, multilayer (>20 layers) films demonstrate enhanced damage resistance with thresholds exceeding 10 MW/cm^2^ [45], yet their nonlinear absorption coefficients are significantly reduced, requiring pump powers >10 mW to initiate mode-locking. In contrast, few-layer (3–10 layers) MoS_2_ achieves a practical balance with mode-locking thresholds in the range of 1–2 mW while maintaining stable operation over extended periods (>100 h) under ambient conditions [41,45]. Beyond chemical stability, interfacial adhesion and mechanical durability are vital for reliable integration. Studies indicate that monolayer MoS_2_ adhesion on SiO_2_ is governed by van der Waals forces and atomic flatness, with optimized interfaces even demonstrating self-healing capabilities under minor deformation. Furthermore, surface modifications have been shown to reduce wear rates by approximately 40% while maintaining a stable friction coefficient [46]. These findings underscore the importance of interfacial engineering in preventing mechanical exfoliation and thermal delamination under intense laser irradiation.

### 2.2. Mainstream Preparation Methods for MoS_2_ SAs

The preparation process of MoS_2_ SAs directly determines their layer distribution, lateral dimensions, film uniformity, and nonlinear optical properties, which ultimately control the output performance and operational stability of mode-locked lasers. In recent years, with the continuous advancement of two-dimensional material fabrication technologies, the preparation of MoS_2_ SAs has evolved from traditional mechanical exfoliation into a diversified technical system integrating scalable manufacturing, precise regulation, and high-performance optimization.

**Figure 2:**
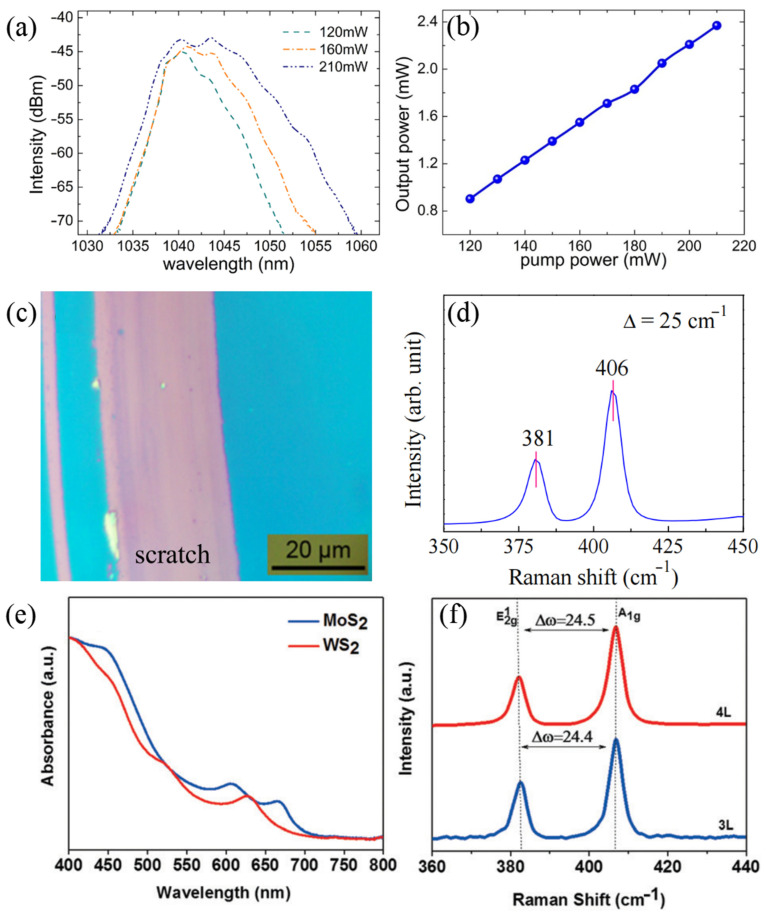
(**a**) Optical spectra of the mode-locked fiber laser at pump powers of 120 mW, 160 mW, and 210 mW; (**b**) Output power versus pump power curve showing a linear relationship Reprinted from Ref. [47]; (**c**) Optical image of a continuous MoS_2_ thin film grown on SiO_2_/Si substrate via the Chemical Vapor Deposition method, demonstrating uniform coverage over a large area; (**d**) Raman spectrum of the CVD-grown MoS_2_ film, exhibiting characteristic E^1^_2g_ and A_1g_ vibrational modes with a frequency difference of 25 cm^−1^, indicative of multilayer MoS_2_; Reprinted from Ref. [48]; (**e**) UV-vis absorption spectra of the MoS_2_ and WS_2_ suspensions. (**f**) Raman spectra of MoS_2_ nanosheets spin-coated onto Si^2+^/SiO_2_ substrates. Reprinted from Ref. [49].

The nonlinear optical performance of MoS_2_ SAs is intrinsically linked to their preparation processes and microstructures. Experimental data demonstrate that high-quality MoS_2_ devices enable stable mode-locked operation. For instance, as shown in Figure 2a, under pump powers ranging from 120 to 210 mW, the central wavelength of the output spectrum remains stable at approximately 1045 nm with a symmetrical profile. Moreover, the linear relationship between output and pump powers, depicted in Figure 2b, confirms that the laser maintains stable mode-locking without multi-pulse or Q-switched instabilities across a broad power range [47]. These metrics validate the capability of high-quality MoS_2_ to drive long-term stable ultrafast lasers.

Mechanical exfoliation remains the most fundamental technique for obtaining high-quality MoS_2_ nanosheets. Recent advancements in dry transfer methods have significantly reduced surface contamination to below 1 nm and improved the successful transfer rate to over 85% [50,51]. This method yields single-layer MoS_2_ with high crystal quality, low defect density [52], and carrier mobilities exceeding 30 cm^2^/V·s, making it well-suited for fundamental physical research. However, its inherent limitations, including restricted flake sizes and uncontrollable layer distribution, preclude its use in scalable manufacturing.

Chemical vapor deposition serves as the core technology for scalable fabrication. CVD-grown MoS_2_ films exhibit continuous and uniform coverage over large areas without apparent voids or cracks, as evidenced by the optical image in Figure 2c, which shows a continuous MoS_2_ thin film uniformly covering an SiO_2_/Si substrate. Raman spectroscopy confirms the structural integrity of the multilayer films, and the corresponding spectrum in Figure 2d displays characteristic E^1^_2g_ and A_1g_ vibrational modes with a frequency difference of approximately 25 cm^−1^, indicative of multilayer MoS_2_ [48]. Through process optimization, CVD-prepared films can achieve damage thresholds exceeding 200 MW/cm^2^, satisfying the requirements for high-power mode-locked lasers [53,54]. Nevertheless, CVD is constrained by high equipment costs, complex parameter tuning, and the susceptibility of transferred films to wrinkles and mechanical damage.

Liquid-phase exfoliation and solution processing offer low-cost and scalable alternatives. Bulk MoS_2_ is exfoliated via ultrasonication and subsequently deposited onto substrates using spin-coating or drop-casting. Figure 2e,f exhibits the UV-vis absorption spectra of the MoS_2_ suspensions and Raman spectra of MoS_2_ nanosheets spin-coated onto Si^2+^/SiO_2_ substrates [49]. Although the resulting nanosheets are relatively small and possess more crystal defects, composite strategies effectively mitigate these issues. For example, just as an adaptive mask-generating algorithm based on fuzzy set theory can automatically optimize threshold selection for noisy data in interferometry [55,56], the incorporation of MoS_2_ into a polymer matrix can effectively compensate for structural defects in exfoliated nanosheets. Nonlinear transmission measurements typically yield a saturation intensity of 0.35 MW/cm^2^ and a modulation depth of 35.4% [49]. Researchers have also developed composite films like MoS_2_/PVA and MoS_2_/SiO_2_ to further improve stability and performance [56,57]. This approach allows for flexible tuning of film thickness and optical properties, facilitating the mass production of cost-effective mode-locked devices, despite potential solvent-induced substrate degradation [58].

Beyond conventional methods, micro–nano fabrication and transfer technologies have advanced device integration, providing important process support for high-quality SAs [59,60]. Current research highlights heterostructure construction and doping modification. Sandwich heterostructures utilizing hexagonal boron nitride and graphene effectively regulate carrier transport, thereby enhancing nonlinear optical performance and operational stability [61]. Furthermore, doping with metallic or non-metallic elements optimizes the band structure and carrier concentration, enabling precise control over key parameters such as modulation depth and saturation intensity.

Comparative analyses underscore the distinct advantages of different preparation routes. While mechanically exfoliated microfiber devices typically exhibit modulation depths around 2.82%, CVD-grown films achieve modulation depths between 5% and 10% with significantly reduced saturation intensities [62]. Additionally, embedding MoS_2_ nanosheets in a PVA matrix can elevate the damage threshold to 1.2 GW/cm^2^, representing a fivefold improvement over pristine films [63]. The superior performance of CVD stems from precise kinetic control during growth, enabling centimeter-scale continuous films with defect densities as low as 10^10^ cm^−2^ [64,65,66]. In contrast, mechanically exfoliated flakes are generally smaller than 10 μm with higher edge defect densities. Moreover, in situ doping during CVD can tune carrier concentrations to 10^13^ cm^−2^, enhancing nonlinear absorption coefficients and suppressing thermal accumulation from non-radiative recombination under high-power irradiation. Consequently, CVD not only facilitates scalable production but also fundamentally enhances the damage tolerance of SAs through optimized crystal quality.

Table 2 summarizes the trade-offs among crystal quality, layer-number control, and cost-effectiveness for various preparation methods. Mechanical exfoliation yields high-quality monolayer or few-layer MoS_2_ but suffers from extremely low production rates, rendering it inadequate for scalable applications. In contrast, chemical vapor deposition enables large-area controllable growth; however, its direct integration into all-fiber devices is hindered by complex transfer processes and stringent high-temperature requirements. Liquid-phase exfoliation and hydrothermal synthesis have emerged as mainstream strategies for large-scale production due to their low cost and operational simplicity, yet they face challenges regarding non-uniform lateral size distribution and restacking issues. Notably, fiber-integrated deposition facilitates the direct fusion of materials with optical fibers, significantly enhancing system compactness and stability despite a limited interaction length. Consequently, the optimal synthesis strategy must be tailored to specific laser performance metrics and fabrication conditions in practical applications.

### 2.3. Performance Characterization Techniques for MoS_2_ SAs

Performance characterization is essential for evaluating the mode-locking potential of MoS_2_ SAs and optimizing their fabrication processes. Current characterization methodologies are mature and can be systematically categorized into linear, nonlinear, and stability assessments, providing a scientific basis for device design and process refinement.

Linear characterization primarily evaluates the fundamental optical properties and crystal structure of the material. Transmission spectroscopy determines the optical absorption characteristics and operational wavelength range, while also providing preliminary insights into layer distribution. Raman spectroscopy serves as the core technique for layer identification [68]; the precise determination of layer thickness and crystal defect density relies on analyzing the frequency shift and intensity ratio of characteristic peaks [69]. Furthermore, atomic force microscopy acts as a complementary tool to visually assess surface morphology, thickness, and film uniformity.

Nonlinear characterization is critical for assessing mode-locking capabilities by measuring saturable absorption properties and carrier dynamics. The Z-scan technique is the standard method for determining third-order nonlinear optical coefficients [70]. By analyzing transmittance variations under different incident intensities, this technique accurately calculates the nonlinear absorption coefficient and saturation intensity, effectively distinguishing between saturable and reverse saturable absorption mechanisms [71]. For instance, mechanically exfoliated monolayer MoS_2_ at 532 nm exhibits a nonlinear absorption coefficient of −1.5 × 10^−12^ m^2^/W, a saturation intensity of approximately 80 MW/cm^2^, and a modulation depth between 5% and 8% [72]. Pump-probe spectroscopy is utilized to measure carrier recovery times, typically revealing a bi-exponential decay. The fast component corresponds to carrier-phonon scattering, while the slow component relates to exciton recombination [73]. These time constants directly determine the theoretical limit of the mode-locked pulse duration [74,75], providing a basis for pulse width optimization [76]. Moreover, integrating the SA into a laser cavity allows for the direct evaluation of practical mode-locking performance, such as the startup threshold and pulse width. For example, CVD-grown MoS_2_ has demonstrated pulse widths as short as 120 fs, a repetition rate of 25 MHz, and a mode-locking threshold of merely 50 mW [77,78,79]. With the advancement of fiber-integrated devices, testing for evanescent field interactions, end-face coupling, and polarization stability has also become integral to comprehensive evaluation [80,81].

Stability characterization evaluates the long-term operational reliability of the SA under practical conditions. This includes long-term operation tests to monitor pulse parameter variations over time, as well as environmental tolerance tests assessing performance under varying temperatures and humidity levels. Polarization tolerance is another crucial metric; experimental results indicate that the modulation depth fluctuation of MoS_2_ SAs remains below 5% across different polarization states, demonstrating excellent stability [82,83]. Overall, this systematic characterization framework provides quantitative guidance for process optimization and establishes unified standards for evaluating the practical application of MoS_2_ in mode-locked fiber lasers.

## 3. Optimized MoS_2_ Fiber Lasers

### 3.1. Pulse Performance Optimization

As a representative two-dimensional transition metal dichalcogenide, the application of MoS_2_ in mode-locked fiber lasers has evolved from initial proof-of-concept demonstrations to a new phase focused on in-depth performance optimization and precise mechanistic regulation. Fundamental research on ultrafast nonlinear pulse dynamics provides essential theoretical support for the generation and manipulation of ultrashort pulses [84]. The observation and analysis of complex pulse behaviors, such as soliton molecules and bound solitons, offer critical insights into the pulse formation mechanisms within MoS_2_-based lasers [85,86]. In addition, investigations into nonlinear dynamical phenomena, including vector solitons and domain wall solitons, provide valuable strategies for optimizing pulse stability in MoS_2_ systems [87]. Building upon these foundations, a profound understanding and precise manipulation of the intrinsic physical mechanisms of MoS_2_ SAs are imperative for overcoming performance bottlenecks and advancing device practicality.

To address the practical requirements of ultrafast fiber lasers, current optimization efforts for MoS_2_-based mode-locked lasers primarily focus on pulse compression, energy scaling, and enhanced stability. Significant breakthroughs in high-performance pulse generation have recently been achieved through the regulation of intrinsic material properties and the management of intracavity dynamics. Moreover, optimizing the laser cavity architecture, dispersion management, and polarization control is critical for realizing superior mode-locked outputs. Rational cavity design and dynamic balance mechanisms are essential to fully exploit the nonlinear modulation capabilities of MoS_2_ SAs [88,89,90,91].

Mode-locked fiber lasers utilizing MoS_2_ SAs exhibit typical soliton mode-locking dynamics. The output spectrum features a central wavelength of 1557 nm and a 3 dB bandwidth of 6 nm, accompanied by distinct Kelly sidebands on both sides. This spectral profile indicates a balance between intracavity dispersion and nonlinearity, providing the necessary spectral foundation for pulse compression [92]. In the time domain, a hyperbolic secant fit to the autocorrelation trace yields a pulse width of 780 fs. This value surpasses the picosecond-level pulse widths typically observed in early two-dimensional material-based mode-locked lasers, confirming the efficient ultrashort pulse shaping capability of MoS_2_ SAs. Moreover, frequency-domain stability tests reveal a signal-to-noise ratio of 65 dB at the fundamental repetition rate, demonstrating high stability and low noise characteristics suitable for reliable long-term operation. This high SNR reflects suppressed amplitude fluctuations resulting from the fast carrier recovery dynamics of MoS_2_, which effectively damps relaxation oscillations and stabilizes the pulse train against environmental perturbations.

Intracavity parameter tuning significantly influences pulse performance optimization. The spectral bandwidth and pulse duration can be tailored through careful adjustment of the net cavity dispersion and SA parameters. This synchronous spectral broadening and pulse compression reflect the coupling mechanism among intracavity dispersion management, polarization state, and material nonlinear modulation. Optimizing intracavity loss and dispersion distribution can further enhance the nonlinear response of MoS_2_, thereby facilitating extreme pulse compression [93].

**Figure 3:**
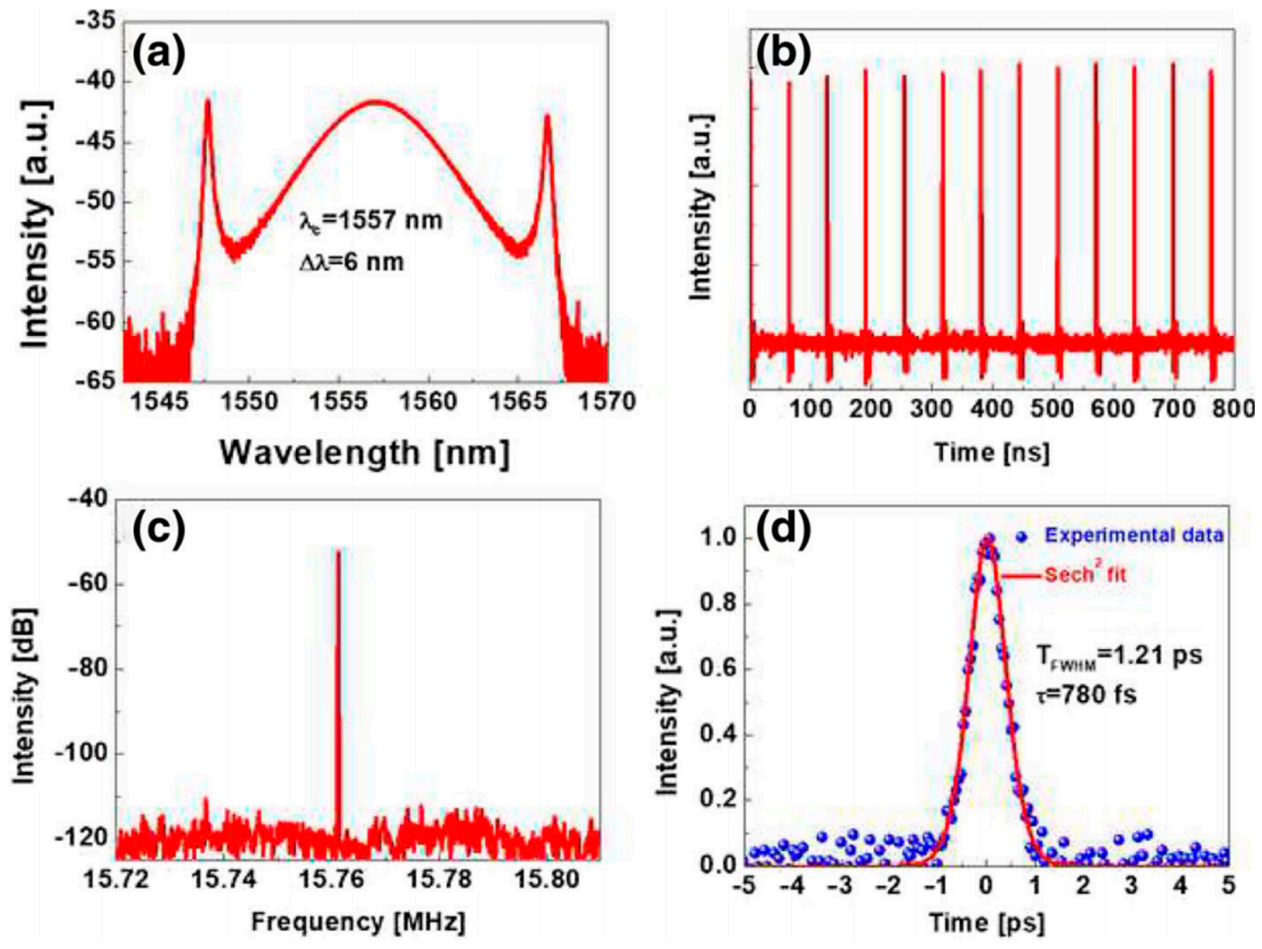
Typical characterization of mode-locked fiber laser pulses based on the MoS_2_/SiO_2_ SA. The optical spectrum in panel (**a**) displays a central wavelength of 1557 nm with a 3 dB bandwidth of 6 nm and distinct Kelly sidebands. Panel (**b**) presents the pulse train in the time domain showing stable operation. The radio frequency spectrum at the fundamental repetition rate exhibits a high signal-to-noise ratio, confirming the stability of the mode-locking operation as shown in panel (**c**). Panel (**d**) illustrates the autocorrelation trace fitted with a sech ^2^ profile, indicating a pulse duration of 780 fs. Reprinted from Ref. [92].

**Figure 4:**
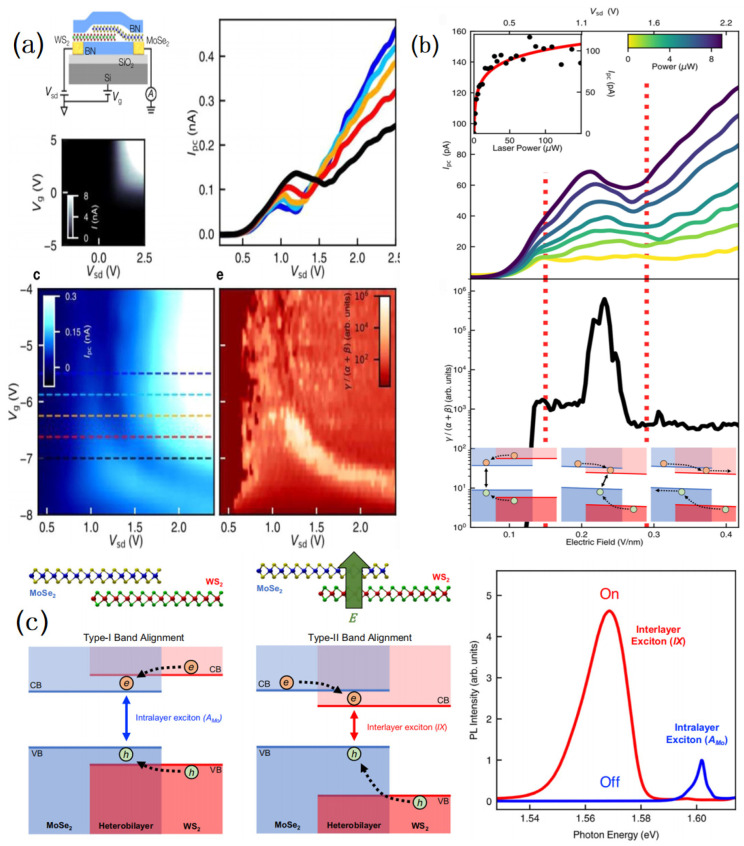
Ultrafast carrier dynamics and interlayer exciton mechanism in the MoS_2_/WS_2_ heterostructure SA. (**a**) Gate-tunable photocurrent response; the saturation at high optical power confirms the nonlinear absorption essential for mode-locking. (**b**) Bias-dependent PL spectra; the emergence of a low-energy emission peak under bias signifies interlayer exciton (IX) formation, with the intensity ratio γ/(α + β) correlating with modulation depth. (**c**) Physical mechanism schematic: the type-II band alignment drives ultrafast interfacial charge transfer, forming spatially indirect IXs that suppress dielectric screening and enable sub-picosecond exciton recombination t(IX) ≈ 1.2 ps. This ultrafast relaxation mechanism provides sufficient modulation speed to sustain the 780-fs pulses observed in Figure 3d, directly linking the heterostructure’s electronic structure to its mode-locking performance. Reprinted from Ref. [97].

The ultimate limit of pulse compression is primarily governed by the carrier relaxation dynamics of the SA. Pump-probe experiments typically reveal that few-layer MoS_2_ possesses ultrafast carrier recovery characteristics with relaxation times ranging from 200 to 500 fs [20]. This sub-picosecond response serves as the fundamental basis for its ability to support ultrashort pulse generation. This sub-picosecond response enables MoS_2_ to effectively filter continuous background light and achieve rapid modulation of ultrashort pulses. Furthermore, constructing van der Waals heterostructures, such as MoS_2_/graphene, can further accelerate the carrier recombination process through interfacial charge transfer mechanisms [94]. Experimental data indicate that the carrier relaxation time in such heterostructures can be reduced to below 500 fs, thereby enhancing the nonlinear modulation depth and suppressing pulse broadening [95,96]. Notably, specifically in the type-II band alignment in the MoS_2_/WS_2_ heterostructure drives ultrafast interfacial charge transfer (<100 fs), forming spatially indirect interlayer excitons (IXs). The suppressed dielectric screening in these IXs accelerates radiative recombination to t(IX) ≈ 1.2 ps, a timescale sufficiently fast to enable the stable generation of 780-fs mode-locked pulses (Figure 3d). This direct link between electronic structure and pulse performance is experimentally verified by the bias-dependent PL and carrier dynamics shown in Figure 4.

#### 3.1.1. Pulse Width Compression and Femtosecond Pulse Generation

The ultimate limit of pulse compression directly reflects the synergistic efficiency between the nonlinear response speed of the SA and intracavity dispersion management [21,98]. To overcome the picosecond-level physical limitations, researchers typically integrate MoS_2_ SAs into anomalous dispersion cavities with fine tuning via dispersion-compensating fibers [99]. Wang et al. demonstrated the broadband saturable absorption of few-layer MoS_2_ from the visible to near-infrared band, confirming its potential as an effective SA for ultrafast pulse generation [21]. This performance stems from precise compensation of third-order dispersion using dispersion-compensating fibers, which minimizes pulse pedestal formation and allows the MoS_2_ saturable absorber to operate near its transform-limited condition. Concurrently, subsequent work achieved femtosecond pulse outputs in the anomalous dispersion regime through synergistic optimization of intracavity dispersion and MoS_2_ SAs [98,99]. This result not only verifies the sub-picosecond ultrafast carrier relaxation time of MoS_2_ but also demonstrates that it has surpassed the performance bottleneck of traditional semiconductor SA mirrors [100,101]. In addition, the long-term stability of mode-locked operation based on MoS_2_ SAs has been experimentally verified; for instance, a CVD-grown multilayer MoS_2_-based SA demonstrated robust and stable mode-locking characterized by a high RF signal-to-noise ratio of approximately 62 dB [11], confirming the feasibility of MoS_2_ SAs for practical applications.

#### 3.1.2. Enhancement of Single-Pulse Energy and Output Power

The core of breaking through power bottlenecks lies in increasing the optical damage threshold of MoS_2_ SAs. Constructing MoS_2_-polymer composite films or employing microfiber evanescent field integration structures can significantly enhance thermal dissipation and laser damage resistance [102]. In polymer composites, the matrix acts as a heat spreader with higher thermal conductivity than air-exposed flakes, reducing local temperature rise per unit absorbed energy; in microfiber configurations, the extended interaction length lowers peak intensity on the MoS_2_ layer for a given average power, distributing thermal load over a larger area. Recent studies confirm that lasers based on MoS_2_ heterostructure SAs can operate stably under high-power pumping. For instance, a Tm:CALGO laser utilizing a MoS_2_ SA achieved an average output power of 1.44 W with a high fundamental repetition rate of 131.6 MHz, corresponding to a single-pulse energy of ~10.9 nJ [103]. It is worth noting that, unlike the typical strategy of increasing pulse energy by lowering the repetition rate in long cavities, this result demonstrates the capability of MoS_2_ SAs to sustain high average power and moderate pulse energy in a high-repetition-rate regime, although the pulse duration was in the picosecond range (~924 ps) due to the Q-switched mode-locking operation. These high-power compatible designs optimize the light–matter interaction length, suppressing harmful nonlinear effects while improving energy extraction efficiency [103,104].

From the perspective of micromechanics and damage tolerance, the power handling capacity of SAs depends not only on the intrinsic bandgap structure of the material but also on its microscopic defects and interfacial stress distribution. Incorporating recent advances in multiscale modeling, finite element stress simulations of the MoS_2_-substrate interface can accurately predict damage initiation thresholds and failure modes [105]. Both experimental data and simulation results indicate that polymer-encapsulated MoS_2_ SAs can achieve damage thresholds of 1.2 GW/cm^2^, representing an approximately fivefold increase compared to pristine MoS_2_ films [64]. The physical essence of this enhancement is that the polymer matrix acts as a stress buffer, dispersing local high stresses generated by photothermal effects, and restricts the degrees of freedom of the MoS_2_ layer to inhibit crack propagation. This matrix-protection-based damage tolerance design offers an effective engineering solution to the thermal lensing and ablation challenges faced by two-dimensional materials under high-power laser irradiation.

#### 3.1.3. Stability Optimization and Environmental Tolerance

The self-starting capability and startup threshold of mode-locking directly determine device practicality. Optimizing SA parameters and dynamically controlling intracavity polarization can significantly lower the mode-locking threshold and enhance operational robustness [106,107]. Lower thresholds are achieved when the saturable absorber’s modulation depth exceeds the total cavity loss margin, while polarization control aligns the intracavity birefringence with the saturable absorber’s anisotropic absorption axis, maximizing effective nonlinear modulation per round trip. Long-term operational stability and environmental adaptability are fundamental metrics for laser practicality. Experimental data demonstrate that lasers utilizing MoS_2_ and h-BN heterostructure SAs exhibit output power fluctuations below 2.5% after eight hours of continuous operation, with energy stability improving by nearly 40% compared to traditional MoS_2_ SAs. This stability advantage originates from the suppression of photothermal effects and the passivation of interfacial defect states by the heterostructure [31,108]. Furthermore, the comprehensive review by Woodward et al. has systematically sum-marized the nonlinear optical properties of few-layer MoS_2_SAs and their integration strategies in short-pulse laser systems, laying a theoretical foundation for subsequent stability optimization and environmental tolerance studies [11].

Collectively, these results validate both the long-term reliability and ultrashort pulse generation capability of MoS_2_ SAs. On one hand, lasers utilizing MoS_2_/h-BN heterostructure-SAs maintain output power fluctuations within 2.5% after eight hours of continuous operation, confirming effective environmental isolation and industrial-grade stability. On the other hand, Wang et al. achieved 620 fs pulse widths in the anomalous dispersion regime through synergistic optimization of intracavity dispersion and MoS_2_ SAs [21], underscoring the material’s advantage in overcoming the pulse compression limits of traditional semiconductor SA mirrors.

### 3.2. Regulation of Nonlinear Absorption Characteristics

**Figure 5:**
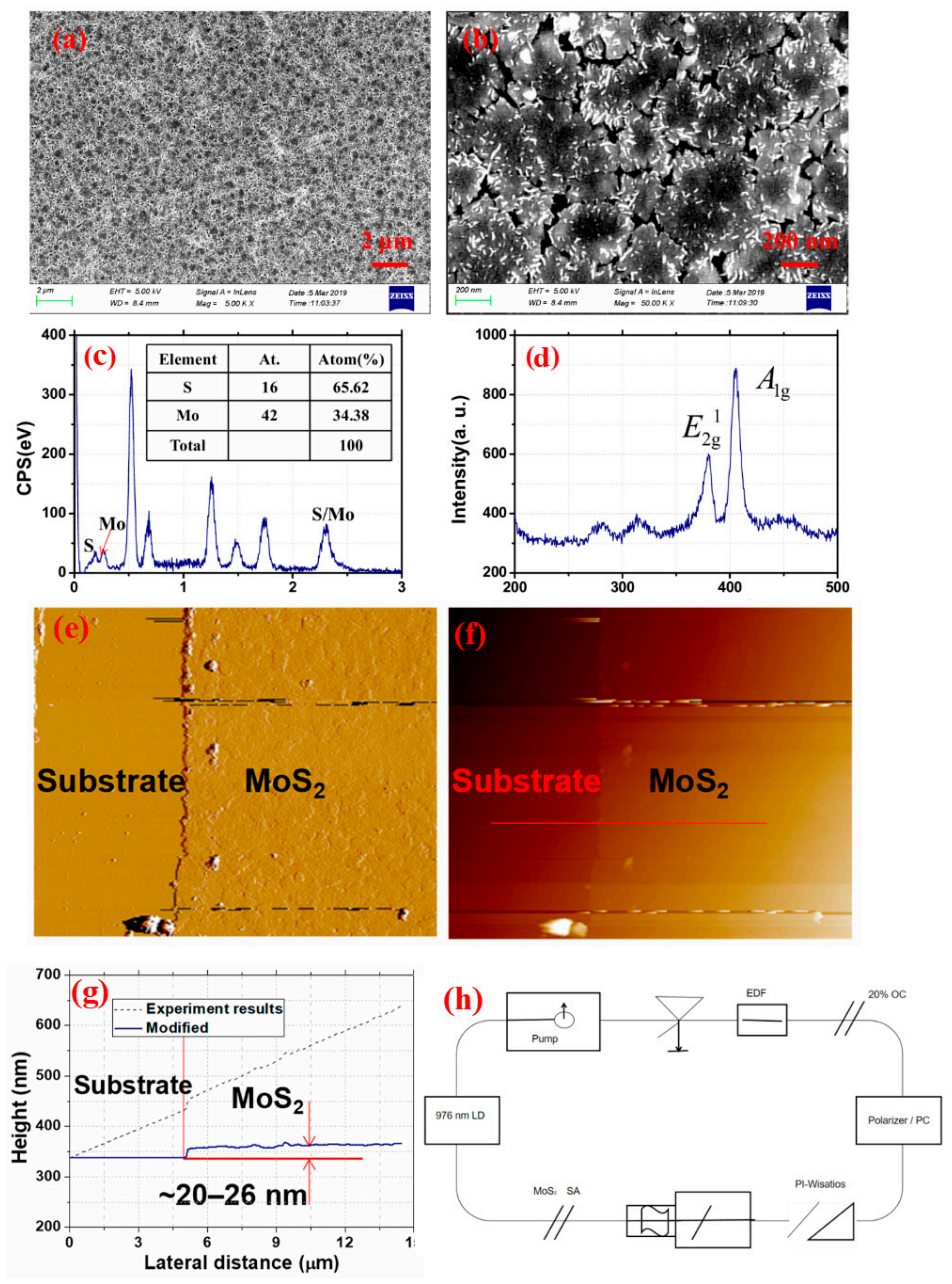
Structural characterization of the prepared MoS_2_ film and the experimental setup. (**a**,**b**) Scanning electron microscopy images of the MoS_2_ film recorded at different resolutions. (**c**) Energy-dispersive X-ray spectroscopy of the MoS_2_ film. (**d**) Raman spectrum of the MoS_2_ film, exhibiting characteristic E^1^_2g_ and A_1g_ peaks. (**e**,**f**) Atomic force microscopy images of the MoS_2_ film. (**g**) Height profile corresponding to the AFM images, indicating a thickness of approximately 20–26 nm. (**h**) Schematic diagram of the passively mode-locked Yb-doped fiber laser based on the MoS_2_ SA. Panels (**a**–**g**) are reprinted from Ref. [109].

High-quality material preparation is vital for achieving excellent nonlinear absorption properties. As shown in Figure 5, scanning electron microscopy images Figure 5a,b show that the prepared MoS_2_ films display uniform and dense microstructures without macroscopic cracks or agglomeration [109]. This confirms that the liquid-phase exfoliation or deposition processes ensure large-area continuity, providing a uniform medium for subsequent optical interactions. Quantitative analysis via energy-dispersive X-ray spectroscopy Figure 5c reveals a molybdenum-to-sulfur atomic ratio near the theoretical 1:2 stoichiometry, indicating high phase purity and minimal impurities. Raman spectroscopy of Figure 5d verifies the crystal quality, revealing distinct in-plane and out-of-plane vibrational modes characteristic of the 2H phase with high crystallinity.

Film thickness is a key geometric parameter determining the strength of the nonlinear optical response. As directly evidenced by the atomic force microscopy images and the corresponding height profile Figure 5e–g, atomic force microscopy measurements indicate a step height of around 20 to 26 nm, corresponding to a multilayer structure. This thickness range ensures sufficient light–matter interaction length to generate significant nonlinear effects while avoiding excessive transmittance losses. The experimental setup for the mode-locked laser Figure 5h uses a compact all-fiber ring cavity pumped by a 980 nm laser diode. Integrating the high-quality MoS_2_ film directly into the cavity leverages its nonlinear absorption to initiate and sustain mode-locking, laying the hardware foundation for high-performance ultrafast laser output [109].

The nonlinear optical properties of two-dimensional transition metal dichalcogenides offer the physical basis for passive mode-locking [110]. The nonlinear absorption behavior of MoS_2_ is mainly governed by ultrafast carrier relaxation dynamics and multiphoton absorption processes, which directly influence pulse formation, compression capability, and high-power stability [21]. Precisely managing the trade-off between carrier relaxation and nonlinear absorption can effectively improve mode-locking performance and expand its application potential in high-power, narrow-pulse fiber lasers.

#### 3.2.1. Ultrafast Carrier Relaxation Mechanisms

Carrier relaxation in MoS_2_ typically involves two distinct timescales: sub-picosecond fast relaxation and picosecond-to-nanosecond slow relaxation. Research indicates that controlling the layer number or introducing defects can tune the relative contributions of these time constants [95]. For instance, few-layer MoS_2_ exhibits shorter relaxation times of approximately 200 to 500 fs, facilitating narrower pulse compression. This ultrafast response enables the effective elimination of continuous background light to form stable pulse trains [111].

#### 3.2.2. Material Characterization and Experimental Validation

The nonlinear absorption of MoS_2_ primarily manifests as a competition between saturable absorption and reverse saturable absorption. At low optical intensities, the saturable absorption effect dominates, increasing transmittance with intensity. In contrast, at high intensities, nonlinear effects such as two-photon absorption (TPA) may induce RSA. To optimize mode-locking performance, researchers employ doping or heterostructure construction to suppress TPA, thereby broadening the linear dynamic range and maintaining excellent modulation characteristics under high-power conditions [112,113,114].

### 3.3. Breakthroughs in MoS_2_-Based Composite SAs

Single-component MoS_2_ materials often face an intrinsic trade-off between modulation depth and damage threshold. To overcome this contradiction, the construction of van der Waals heterostructures has emerged as the mainstream technical pathway for enhancing mode-locking performance.

**Figure 6:**
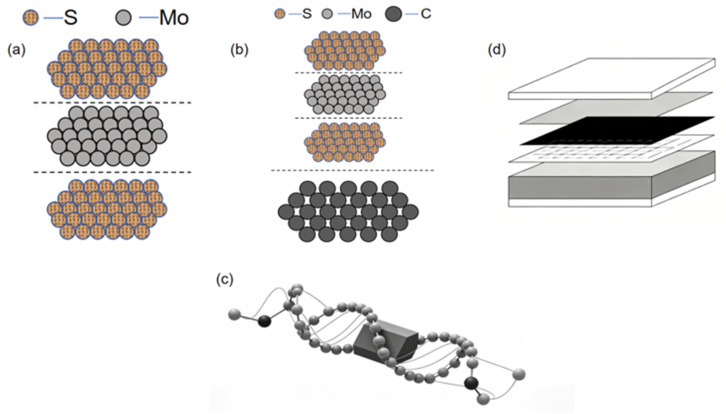
Schematic diagrams of different MoS_2_-based material structures. (**a**) Pristine MoS_2_ atomic structure. (**b**) MoS_2_/Graphene vdW heterostructure. (**c**) MoS_2_ embedded in a polymer matrix. (**d**) Schematic of a plasmonic-enhanced MoS_2_ composite structure.

Despite the significant nonlinear optical response of intrinsic MoS_2_, its performance remains limited by the intrinsic band structure and interlayer coupling, particularly regarding modulation depth, damage threshold, and carrier relaxation time [105]. To address these limitations, composite structural designs based on band engineering and interfacial modulation have become core strategies for enhancing MoS_2_-based SAs [115,116,117,118]. As illustrated in Figure 6, mainstream composite configurations include pristine MoS_2_ as a reference, the layered stacking of MoS_2_/graphene van der Waals heterostructures, MoS_2_ embedded within a polymer matrix [119], and hierarchical plasmon-enhanced MoS_2_ architectures. This section reviews recent advances in synergistically optimizing mode-locking performance through heterostructures, polymer composites, and plasmonic enhancement.

Different design strategies yield varying degrees of performance enhancement. For MoS_2_/graphene systems, Sun X.D. et al. observed that interfacial charge transfer significantly amplifies the nonlinear optical response [96]. The modulation depth of this heterostructure increased by approximately 30% compared to pristine MoS_2_, with carrier relaxation times reduced to below 500 fs. In MoS_2_/BP systems, Xue Y. et al. utilized the narrow bandgap of BP to extend the operational wavelength [120]. This heterostructure not only achieved Q-switching at 2.0 μm but also nearly doubled the nonlinear absorption coefficient relative to pristine BP, Furthermore, ultrafast carrier dynamics studies of BP/MoS2 heterostructures have revealed rapid charge transfer and efficient carrier relaxation processes that underpin the feasibility of such composite SAs for high-speed photonic applications [121]. These findings demonstrate that composite structures can resolve the performance bottlenecks of single materials in specific spectral bands or under high-power conditions.

#### 3.3.1. Two-Dimensional Material Heterostructures

The formation of conventional and dissipative solitons relies on the synergy of dispersion, nonlinearity, gain, and loss. Heterostructure design can further suppress multi-soliton competition and enhance pulse stability [122,123]. The MoS_2_/graphene heterostructure combines the strong light–matter interaction of MoS_2_ with the ultra-broadband response of graphene. Interfacial charge transfer effectively modulates the Fermi level, optimizing saturable absorption and improving mode-locking stability [24,124,125,126]. Experimental data indicate that lasers utilizing this heterostructure achieve stable mode-locking with pulse widths as short as −837 fs at 1.55 μm, with a modulation depth of 12.4% [127].

For mid-infrared applications, the MoS_2_/BP heterostructure leverages the tunable bandgap of BP for efficient mode-locking at 2 μm [120]. MoS_2_ serves as a protective layer to prevent the environmental degradation of BP. Moreover, the favorable band alignment facilitates photogenerated carrier separation, accelerating carrier relaxation and enabling narrower pulses. The nonlinear absorption coefficient of this heterostructure is nearly twice that of pristine BP [128]. Heterostructures combining MoS_2_ with other transition metal dichalcogenides, such as WS_2_ or MoSe_2_, also exhibit superior performance. Variations in band structure and carrier mobility allow for band matching and synergistic optimization of carrier dynamics, further enhancing nonlinear optical responses [129,130].

#### 3.3.2. MoS_2_/Polymer Composites

Dispersing MoS_2_ nanosheets in polymer matrices, such as polymethyl methacrylate or polyvinyl alcohol, yields mechanically robust and easily integrable composite films. The polymer matrix prevents nanosheet agglomeration and assists in suppressing background noise through optical limiting effects. Crucially, the high thermal conductivity and mechanical strength of the polymer significantly improve the damage threshold of the SA [74]. Experimental results show that MoS_2_/PVA composite films achieve a damage threshold of 1.2 GW/cm^2^, representing a fivefold increase over pristine MoS_2_ films [63]. These composites maintain excellent nonlinear absorption while offering flexibility and processability for fiber device integration, enabling stable nanosecond pulse generation at 1.0 μm with superior long-term operational stability.

#### 3.3.3. MoS_2_/Metal Nanostructure Composites

Integrating MoS_2_ with metallic nanostructures, such as gold or silver nanoparticles, exploits surface plasmon resonance to enhance light–matter interactions [114]. The SPR effect generates intense localized electromagnetic fields, significantly amplifying the nonlinear absorption coefficient of MoS_2_. Studies indicate that MoS_2_/Au nanoparticle composites exhibit a nonlinear absorption coefficient approximately one order of magnitude higher than pristine MoS_2_ [131]. Lasers based on these plasmonic composites achieve mode-locking at reduced pump thresholds. Notably, the SPR effect modulates carrier dynamics in MoS_2_, accelerating carrier relaxation and facilitating the generation of narrower pulses [132].

**Figure 7:**
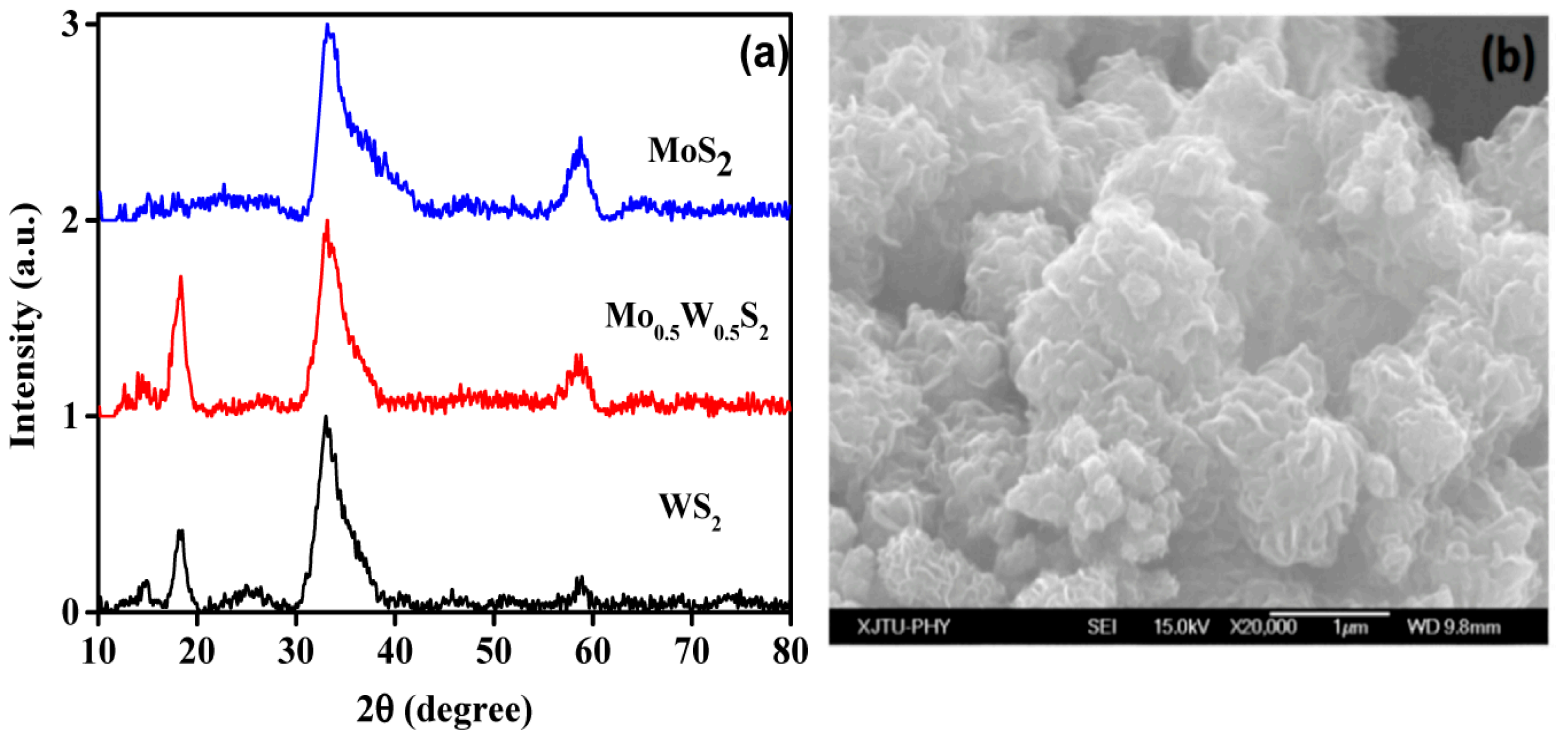
Structural characterization of Mo_0.5_W_0.5_S_2_ alloy as a typical TMD alloy system. (**a**) X-ray diffraction patterns showing the lattice constant variation between MoS_2_, WS_2_, and the alloy. (**b**) SEM image displaying the flower-like microstructure of the synthesized alloy. Reprinted from Ref. [133].

As illustrated in Figure 7, the successful synthesis of Mo_0.5_W_0.5_S_2_ alloys not only validates the feasibility of tuning lattice structures via alloying but also exhibits a distinctive hierarchical flower-like morphology. This unique architecture synergistically boosts nonlinear optical performance. The flower-like structure significantly increases the specific surface area, providing abundant active sites for light–matter interactions. Concurrently, lattice constant variations confirmed by X-ray diffraction effectively modulate the band structure and optimize carrier dynamics. Compared to pristine MoS_2_, this alloyed system leverages synergistic elemental effects to substantially enhance the nonlinear absorption coefficient and accelerate carrier relaxation. These attributes are critical for achieving low-threshold, ultrashort-pulse laser generation. Such structural characteristics further highlight the core value of transition metal dichalcogenide alloying strategies in overcoming the performance bottlenecks of single materials, offering novel structural paradigms for developing highly efficient SAs for ultrafast lasers [133].

Collectively, constructing heterogeneous and composite structures can effectively overcome the intrinsic limitations of pristine MoS_2_, achieving synergistic performance enhancement and providing an effective pathway for developing high-performance MoS_2_ SAs. Furthermore, integrating dispersion management, polarization optimization, and intrinsic parameter modification of SAs enables multidimensional improvements in mode-locking performance, offering universal strategies for the design of high-performance ultrafast lasers [134,135,136]. Future research should focus on exploring advanced hybrid architectures and composite architectures and elucidating their underlying physical mechanisms to achieve superior mode-locking performance.

Table 3 summaries the performance comparison of different MoS_2_-based composite SAs. The data highlights that constructing composite structures effectively tunes nonlinear optical properties. Specifically, MoS_2_/Graphene heterostructures offer ultrafast response for ultrashort pulse lasers, while MoS_2_/BP composites extend operation to the mid-infrared (2.0 µm). Polymer encapsulation enhances environmental stability for all-fiber integration, and plasmonic nanostructures significantly reduce saturation intensity (<10 MW/cm^2^), facilitating low-threshold mode-locking. These strategies demonstrate that interface engineering can overcome the bottlenecks of pristine MoS_2_.

## 4. Application Comparison and Prospects of Carbon Nanotube and MoS_2_ SAs

Carbon nanotubes and molybdenum disulfide serve as representative nonlinear optical materials, both demonstrating unique advantages as SAs in mode-locked fiber lasers. However, they exhibit significant differences in physical mechanisms, performance parameters, and application scenarios. From a material perspective, CNTs have been a mainstream choice for passive mode-locking since the 1990s due to their exceptional mechanical strength, thermal conductivity, broadband absorption, and mature fabrication processes, making them dominant in industrial high-power applications. In contrast, MoS_2_ has emerged as a prominent two-dimensional material in the post-graphene era. Characterized by its atomic thickness, tunable bandgap, and strong light–matter interaction, it has shown unique potential in ultrafast photonics, particularly in mid-infrared bands and femtosecond pulse generation [137,138].

From a technological evolution perspective, CNT technology has reached maturity with clear device integration pathways and controllable costs. However, constrained by intrinsic nonlinear refractive indices and damage thresholds, CNTs are gradually encountering physical bottlenecks in extreme power or ultrashort pulse scenarios. In contrast, MoS_2_ is currently in a phase of technological breakthrough. Through heterostructure design, polymer composites, and surface plasmon modulation, its performance boundaries are continuously expanding. Although optimization in scalable fabrication and long-term stability is still required, its irreplaceability in emerging application scenarios has become increasingly prominent.

### 4.1. Application Scenarios and Value Comparison Across Different Wavelength Bands

In practical applications of mode-locked fiber lasers, the operating wavelength directly determines the value and feasibility of the application scenarios. MoS_2_ and CNTs exhibit distinct performance characteristics and application prospects at 1.0 µm, 1.5 µm, and 2.0 µm bands.

At the 1.0 µm band, which primarily corresponds to ytterbium-doped fiber lasers for ultrashort pulse generation and micro–nano processing, CNT SAs demonstrate outstanding advantages. Benefiting from mature fabrication processes, they achieve stable picosecond-level mode-locked output and are widely used in fundamental research and medium-power industrial marking [28,139]. The advantage of MoS_2_ SAs lies in their sub-picosecond carrier relaxation time at this band, enabling lasers to surpass the picosecond limit and achieve femtosecond pulse output. For instance, MoS_2_ saturable absorbers based on side-polished fiber or fiber-ended deposition structures have successfully realized passive mode-locking in fiber lasers [140,141]. In precision micro-processing scenarios requiring extreme temporal resolution, MoS_2_ exhibits higher application value than CNTs due to its narrower pulse width and smaller heat-affected zone [132].

At the 1.5 µm band, the standard window for fiber communication systems and a critical region for high-power fiber lasers, CNTs remain the traditional dominant choice. Their high damage threshold and excellent chemical stability make them the preferred solution for high-power industrial processing and long-distance optical communication systems due to their maturity and reliability [142,143]. MoS_2_ faces challenges at this band, primarily its relatively low damage threshold and susceptibility to environmental influences such as oxidation and defect-mediated degradation [144,145]. However, owing to its exceptionally high nonlinear absorption coefficient and layer-tunable bandgap, MoS_2_ provides superior modulation depth and more stable mode-locking states in scenarios demanding extreme pulse quality rather than extreme power. Through composite structures such as MoS_2_-polymer hybrids, MoS_2_ is progressively closing the gap with CNTs in power handling capacity [23,92,146,147,148,149].

At the 2.0 µm band, located in the eye-safe region and corresponding to strong absorption bands of water and organic molecules, it serves as a critical window for biomedicine and gas sensing. Narrow-gap materials such as CNTs and black phosphorus possess intrinsic absorption advantages at this wavelength due to their naturally matched bandgaps. In contrast, the photon energy at 2.0 µm (~0.62 eV) lies well below the direct bandgap of monolayer MoS_2_ (~1.8 eV); therefore, its saturable absorption in this band does not originate from resonant interband transitions, but is instead attributed to defect-/edge-state-mediated sub-bandgap absorption and two-photon absorption processes, as evidenced by giant near-infrared TPA coefficients in monolayer MoS_2_ [22], enhanced TPA via sulfur vacancy engineering [150], picosecond-scale defect-assisted carrier capture dynamics [95], and strain/electric-field tunability of two-photon absorption thresholds [151]. While MoS_2_ does not offer intrinsic superiority over narrow-gap alternatives at 2.0 µm, it presents competitive broadband applicability owing to its robust environmental stability, ease of all-fiber integration, and scalable preparation—advantages that complement the higher intrinsic nonlinearity of CNTs and BP. In frontier fields such as optical coherence tomography, minimally invasive surgery, and mid-infrared gas detection, MoS_2_-based SAs thus serve as a viable and practically advantageous option for mode-locked light sources in this band, particularly where device reliability and integration convenience are prioritized [152,153].

### 4.2. Application Evolution of MoS_2_ and CNT SAs in Mode-Locked Lasers

**Figure 8:**
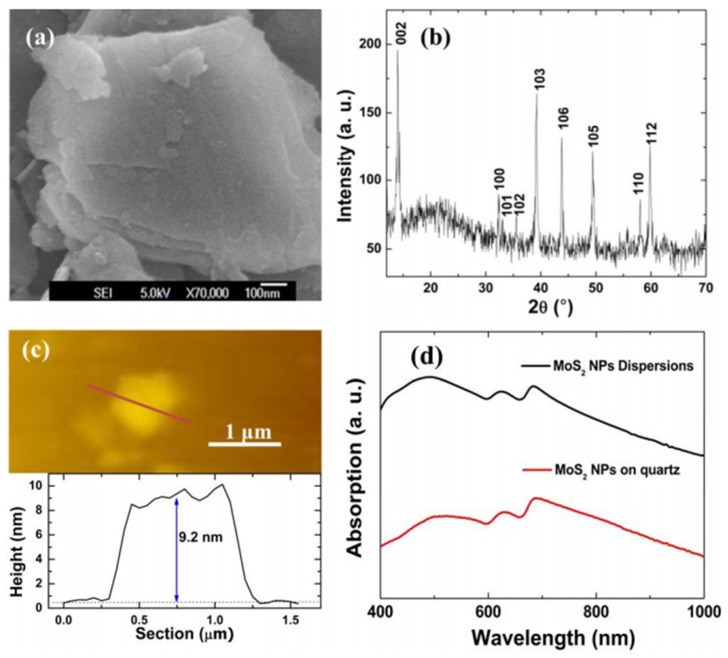
Comprehensive characterization of MoS_2_ nanomaterials. (**a**) Scanning electron microscopy image depicting the layered stacking morphology of the as-prepared MoS_2_ nanoplatelets. (**b**) X-ray diffraction pattern confirming the hexagonal crystal structure of the exfoliated MoS_2_. (**c**) Atomic force microscopy topography and the corresponding height profile, quantitatively revealing a thickness of approximately 9.2 nm for the nanosheet. (**d**) UV-Vis-NIR absorption spectrum showcasing the distinct A and B excitonic absorption peaks at 630 nm and 680 nm, respectively, along with the broadband absorption feature. Reprinted from Ref. [157].

At the core device level of mode-locked fiber lasers, CNTs and MoS_2_ exhibit significant complementarity and competition in physical mechanisms and performance. Specifically for MoS_2_, two-dimensional transition metal dichalcogenides demonstrate unique application potential in nonlinear photonics due to band structure evolution induced by quantum confinement effects. As a typical representative, monolayer MoS_2_ possesses a direct bandgap of approximately 1.8 to 1.9 eV, triggering strong excitonic resonance effects and significant nonlinear optical responses [154]. As shown in Figure 8, comprehensive characterization of the as-prepared MoS2 nanomaterials directly validates these structural and optical properties: the scanning electron microscopy image Figure 8a clearly depicts the layered stacking morphology of the MoS_2_ nanoplatelets, confirming the characteristic two-dimensional layered architecture; the X-ray diffraction pattern Figure 8b confirms the hexagonal crystal structure of the exfoliated MoS_2_, consistent with the highly ordered hexagonal lattice arrangements and the interlayer van der Waals spacing precisely locked at 0.62 nm [155]; the atomic force microscopy topography and corresponding height profile Figure 8c quantitatively reveal a nanosheet thickness of approximately 9.2 nm, indicating a multilayer structure that preserves the van der Waals interlayer stacking while maintaining the nonlinear optical response governed by quantum confinement. Optical measurements indicate excellent broadband absorption from visible to near-infrared regions, with A and B exciton peaks located at 632 nm and 675 nm, respectively, as directly evidenced by the UV-Vis-NIR absorption spectrum Figure 8d, and corresponding absorption coefficients reaching the order of 10^5^. Its ultrafast carrier dynamics are dominated by defect-assisted Auger recombination mechanisms, with relaxation time constants as low as the sub-picosecond scale [156], providing a physical foundation for efficient femtosecond mode-locking. Notably, a low saturation intensity threshold of 5 to 10 MW/cm^2^ and a modulation depth of 10% to 15% grant it significant competitive advantages in low-power laser systems. These intrinsic physical parameters and microstructural characteristics collectively determine the application potential of MoS_2_ as a high-performance SA.

#### 4.2.1. Physical Mechanisms and Advantages of CNTs and MoS_2_

As a one-dimensional quantum material, the nonlinear optical properties of carbon nanotubes (CNTs) primarily originate from their unique band structure and high electron mobility. In practical applications, CNTs exhibit an exceptionally high modulation depth, typically ranging from 10% to 20%, providing inherent advantages in suppressing continuous-wave lasing and maintaining pulse stability [143]. Moreover, the outstanding mechanical strength and thermal conductivity of CNTs endow them with a high damage threshold, enabling them to withstand high-power laser fluxes at the watt level. Consequently, they have been widely adopted in industrial-grade high-power fiber lasers [158,159]. However, the bandgap of CNTs is strictly constrained by their diameter and chirality. Although broadband absorption can be achieved by mixing tubes of different chiralities, absorption efficiency significantly degrades at specific wavelengths. Furthermore, the nonlinear response speed of CNTs is limited by carrier-phonon scattering processes, typically occurring on the picosecond scale, which makes it challenging to meet the extreme requirements for femtosecond pulse generation [160,161].

In contrast, molybdenum disulfide, as a typical two-dimensional transition metal dichalcogenide, exhibits strongly layer-dependent physical properties. Comprehensive characterizations reveal the correlation between its microstructure and optical characteristics. Morphological and thickness analyses via scanning electron microscopy and atomic force microscopy confirm that exfoliated MoS_2_ nanosheets possess a layered stacking structure with a thickness of approximately 9.2 nm. This few-layer structure induces an indirect-to-direct bandgap transition, thereby generating a strong excitonic effect. Crystallographic analysis via X-ray diffraction displays distinct characteristic peaks, confirming a well-defined hexagonal crystal structure that serves as the foundation for its superior optoelectronic performance. Optically, ultraviolet-visible-near-infrared absorption spectra exhibit pronounced A and B exciton absorption peaks accompanied by broadband background absorption. The direct bandgap in monolayer or few-layer MoS_2_ yields a strong excitonic effect and significant nonlinear optical response, allowing for saturable absorption at low incident optical intensities. This low saturation intensity threshold makes MoS_2_ highly suitable for low-power, high-efficiency mode-locked laser designs [162]. The ultrafast carrier dynamics of MoS_2_—typically characterized by a primary relaxation component in the 200–500 fs range [20,132]—are central to its function as a saturable absorber. This sub-picosecond recovery enables efficient suppression of continuous-wave (CW) background light and rapid nonlinear modulation of ultrashort pulses, which is essential for stabilizing soliton mode-locking and mitigating Q-switching instabilities. Crucially, the observed recovery kinetics are highly sensitive to structural integrity: in pristine exfoliated nanosheets, defect-assisted Auger recombination dominates, enabling near-ideal sub-500 fs response; in contrast, CVD-grown or hybrid-integrated films often exhibit sulfur vacancies and interfacial trap states that introduce slower (ps-scale) decay components, compromising pulse fidelity and promoting Q-switched instabilities. Thus, the intrinsic ~200–500 fs timescale serves as an upper bound, and the actual performance in laser devices depends critically on defect engineering and interface control [134,163]. Additionally, the bandgap of MoS_2_ can be tuned from 1.2 eV to 1.8 eV by adjusting the layer number, offering flexible spectral response ranges from the visible to the near-infrared region [154].

Nevertheless, compared to CNTs, MoS_2_ possesses relatively low thermal conductivity, and its atomic-scale thickness renders it highly susceptible to thermal accumulation and structural damage under high-power laser irradiation [161]. This constitutes the primary physical bottleneck restricting its promotion in extreme high-power industrial applications [164]. Moreover, the fabrication process of MoS_2_ is highly sensitive to environmental conditions, such as environmental instability caused by sulfur vacancy defects. Its long-term stability still requires improvement compared to mature commercial CNT products [161].

Despite the complementary advantages of individual components, MoS_2_/CNT hybrid saturable absorbers face intrinsic limitations that constrain their broader adoption. First, the damage threshold of the composite remains capped by the heterogeneous interface: while CNTs provide thermal conduction pathways, localized Joule heating at MoS_2_-CNT junctions under high repetition rates can trigger irreversible degradation well below the theoretical limit of either constituent. Second, environmental sensitivity persists due to residual sulfur vacancies and adsorbed species at the interface, which act as charge traps and accelerate performance decay under ambient humidity—passivation strategies mitigate but do not eliminate this vulnerability. Third, and most critically, there exists an unavoidable trade-off between modulation depth and insertion loss in hybrid SAs: increasing MoS_2_ loading enhances nonlinear modulation but simultaneously raises linear absorption and scattering losses, reducing overall cavity efficiency; conversely, optimizing for low loss compromises self-starting reliability. This fundamental compromise limits the simultaneous achievement of high-energy pulses and robust operation, distinguishing hybrid SAs from idealized theoretical models.

In summary, CNTs and MoS_2_ possess distinct advantages as SAs. CNTs remain highly competitive in high-power, long-lifespan industrial applications due to their high damage threshold and mature processing. On the other hand, MoS_2_ demonstrates great potential in generating ultrashort pulses, expanding operating wavelength bands, and constructing miniaturized, integrated photonic devices, driven by its ultrafast nonlinear response and tunable band structure. The performance disparities do not represent a simple division of superiority and inferiority but rather point toward distinct technical application scenarios and developmental trajectories.

#### 4.2.2. Composite Structural Design and Interface Engineering

To overcome the thermal damage bottleneck of single materials under high-power laser irradiation, researchers have proposed composite structural design strategies. The core of this strategy lies in utilizing synergistic effects by introducing auxiliary materials with high thermal conductivity or wide bandgaps to compensate for the shortcomings of the host material.

For MoS_2_ composite optimization, mixing MoS_2_ with high-thermal-conductivity graphene or carbon nanotubes can construct efficient electron and phonon transport channels. For instance, MoS_2_/graphene heterostructures not only retain the exceptionally high nonlinear modulation depth of MoS_2_ but also utilize the superior thermal conductivity of graphene to rapidly dissipate heat, significantly elevating the device’s damage threshold. Experiments indicate that while maintaining femtosecond-scale carrier relaxation speeds, such composite materials can withstand incident powers three to five times higher than pristine MoS_2_ films [39,65,146].

Regarding interfacial modification of CNTs, although CNTs possess high intrinsic thermal conductivity, thermal resistance increases during high-density packing. Modifying the CNT surface with polymers or inorganic dielectric layers can optimize their dispersion on substrates and mitigate the formation of localized hot spots [165].

For universal packaging technologies, utilizing microfibers or planar waveguides as carriers to encapsulate two-dimensional materials within low-loss dielectrics like SiO_2_ or Al_2_O_3_ protects the materials from environmental degradation. Additionally, the strong optical field confinement effect of the waveguide reduces the stringent damage threshold requirements of the materials themselves [44,45].

### 4.3. Constraints of Intrinsic Material Properties and Breakthroughs via Process Optimization

The core challenge for CNT SAs lies in the uncontrollability of their physical properties. Due to fabrication limitations, commercial CNTs typically exhibit non-uniform distributions in tube diameter, wall number, and chirality. This non-uniformity directly broadens the bandgap distribution of the SA, thereby affecting the pulse stability and signal-to-noise ratio of mode-locked lasers. However, this challenge is being effectively mitigated through advanced separation and purification technologies. Recent advancements in density gradient centrifugation and gel chromatography have enabled the acquisition of high-purity semiconducting CNTs. Moreover, precisely controlling the alignment density and deposition methods of CNTs effectively suppresses undesirable nonlinear backgrounds, significantly enhancing device performance consistency [166].

As a two-dimensional material, the dangling bonds on the surface of MoS_2_ make it susceptible to erosion by oxygen and water molecules in atmospheric environments, leading to performance degradation over time. This environmental sensitivity is a primary concern for industrial-grade, long-lifespan applications. Addressing this bottleneck, recent research indicates that interface engineering and heterostructure construction are effective solutions [167]. Constructing van der Waals heterostructures by sandwiching MoS_2_ between hexagonal boron nitride layers, or employing atomic layer deposition for surface passivation and encapsulation, can effectively isolate environmental interference and significantly enhance long-term stability. These protection strategies have extended the operational lifespan of MoS_2_ devices in laboratory environments several times over.

Both materials share common challenges. In high-power mode-locking applications, both face damage risks induced by thermal accumulation. Although CNTs possess high thermal conductivity, they are prone to localized ablation under high-flux irradiation, whereas the relatively low thermal conductivity of MoS_2_ makes thermal management even more challenging. To break through this power limit, composite structural design has become a current research hotspot. Heterojunctions combining topological insulators with MoS_2_ can further enhance nonlinear absorption and achieve rapid self-starting, providing new pathways for low-threshold, highly stable mode-locked devices [168]. Analogously, compositing CNTs or MoS_2_ with high-thermal-conductivity polymers or graphene not only utilizes the matrix material for auxiliary heat dissipation but also optimizes nonlinear absorption characteristics through interfacial effects. Such hybrid integration significantly improves the damage threshold of SAs, ensuring the stable operation of high-power ultrafast lasers.

Beyond thermal management, achieving large-area, high-yield manufacturing from laboratory to industrialization remains a shared challenge. Traditional micro-mechanical exfoliation cannot meet mass production demands, while direct growth methods face difficulties in integrating with fiber end faces. This challenge is being overcome by novel micro–nano fabrication technologies. For example, combining roll-to-roll transfer technology with chemical vapor deposition has enabled the preparation of centimeter-scale uniform MoS_2_ films. For CNTs, in situ growth technologies based on fiber end faces are also maturing. These technological advancements are progressively reducing manufacturing costs and driving the transition of both materials from scientific samples to standardized commercial devices.

## 5. Summary and Outlook

### 5.1. Research Status and Core Conclusions

This paper provides a systematic overview of the research progress on MoS_2_-based mode-locked fiber lasers, covering three dimensions: intrinsic material properties, SA construction and characterization, and laser performance. The nonlinear optical properties of MoS_2_ depend heavily on its layer-dependent band structure [169,170]. Current mainstream preparation techniques include mechanical exfoliation, chemical vapor deposition (CVD), and liquid-phase exfoliation [160,171]. Mechanical exfoliation produces high-quality single crystals, CVD shows greater potential for fabricating large-area films with controllable layer numbers, and liquid-phase exfoliation is advantageous for low-cost, large-scale production of dispersions [57,172].

Regarding SA construction, fiber end-face integration, D-shaped fiber side polishing, and microfiber evanescent field coupling are the dominant integration schemes [158,173]. Characterizations via Z-scan and pump-probe techniques confirm that MoS_2_ possesses a low saturation intensity, high modulation depth, and sub-picosecond ultrafast carrier relaxation time, making it a promising candidate for ultrashort pulse generation [163,174,175]. Integrating MoS_2_ SAs into fiber laser cavities has successfully achieved stable mode-locking across the 1.0 μm to 2.0 μm spectral range [176]. These lasers generate ultrashort pulses ranging from picoseconds to femtoseconds and demonstrate distinct control capabilities in complex dynamical mechanisms such as multi-wavelength mode-locking and dissipative soliton resonance [177,178,179]. These results demonstrate the potential of MoS_2_ as a complementary alternative to traditional SESAMs in low-to-medium power ultrafast fiber lasers, while acknowledging that SESAMs remain the dominant solution for high-power commercial applications.

However, current research faces several critical challenges. Few-layer MoS_2_ is prone to environmental degradation under high-power laser irradiation, which limits the long-term stability of devices [144,145,161]. Furthermore, fluctuations in layer uniformity during CVD growth and residual dispersants in liquid-phase exfoliation lead to significant batch-to-batch performance variations in SA devices [160,171]. Additionally, the intrinsically low thermal conductivity of MoS_2_ and the thermal lensing effect at the fiber coupling interface limit further improvements in single-pulse energy [164,165].

### 5.2. Development Trends and Technical Pathways

Based on current research progress and the overall development trends in the two-dimensional materials field, the future evolution of MoS_2_-based mode-locked fiber lasers will focus on four key directions: material engineering, integration processes, performance breakthroughs, and application extensions.

In material engineering, the research focus will shift from single-material systems to van der Waals heterostructures. Stacking MoS_2_ with hexagonal boron nitride or graphene will achieve environmental isolation and carrier dynamics regulation, further optimizing nonlinear absorption characteristics [180,181,182,183]. In integration processes, all-polarization-maintaining fiber integration will become the core direction for industrial-grade applications. Combining this with advanced micro–nano fabrication technologies to directly grow or transfer MoS_2_ onto photonic crystal fibers or integrated photonic chips is expected to enable ultrafast light sources with smaller footprints and higher efficiencies [184,185]. However, realizing these pathways requires overcoming concrete engineering bottlenecks that remain underexplored. First, transferring MoS_2_ onto PM fiber end-faces or side-polished surfaces inevitably introduces asymmetric mechanical stress and interfacial contamination, which degrade the polarization extinction ratio and undermine the fundamental advantage of all-PM architectures [186]; stress-compensated bonding and in situ PER monitoring during fabrication are therefore essential but not yet mature. Second, atomic-layer integrity is highly vulnerable during coating and transfer steps: thermal expansion mismatch between MoS_2_ and polymer/fiber substrates, combined with capillary forces in wet processing, generates localized strain concentrations that induce micro-cracking and irreversible nonlinear performance loss [187,188]. Third, while advanced integration schemes promise enhanced performance, the field lacks standardized protocols for evaluating SA degradation under realistic operational loads; virtually no studies characterize device evolution under continuous intracavity powers exceeding 100 mW over extended durations [10,92,108], making it impossible to qualify MoS_2_ SAs for industrial deployment without establishing community-agreed test standards encompassing accelerated aging, in situ spectral monitoring, and post-mortem morphological analysis [189]. Addressing these specific obstacles is a prerequisite for translating laboratory-scale integration demonstrations into reliable commercial products.

Regarding performance breakthroughs, bandgap engineering approaches such as alloying and strain tuning are expected to extend the operational wavelength of MoS_2_-based SAs into the mid-infrared region beyond 2 μm. Concurrently, combining large-mode-area fibers with novel nonlinear management techniques will address high-power thermal management challenges, achieving high single-pulse energy mode-locked output. In application extensions, MoS_2_-based mode-locked lasers will transition from laboratory research to practical scenarios including biomedical imaging, precision spectroscopy, and optical communications [5,189]. Developing customized two-dimensional material mode-locked lasers tailored to specific application requirements for pulse width, repetition rate, and wavelength will be instrumental in advancing the industrialization of this technology [190].

### 5.3. Industrial Viability

The transition of MoS_2_-based saturable absorbers from laboratory-scale demonstrations to industrial deployment requires a balanced assessment of manufacturing economics, process scalability, and long-term operational stability—dimensions that are often underemphasized in fundamental studies yet critical for practical adoption. Solution-processed approaches, particularly liquid-phase exfoliation, are widely regarded as promising for scalable production due to their inherent throughput potential relative to CVD or mechanical exfoliation [57]. Nevertheless, realizing this scalability hinges on achieving consistent nanosheet thickness and lateral dimensions across production batches, a challenge that has not been fully resolved. Although CVD produces materials with superior crystallinity, its adaptation to roll-to-roll manufacturing remains constrained by transfer-induced defects and substrate compatibility limitations, as evidenced in wafer-scale growth studies [191].

In terms of industrial scalability, the principal bottleneck resides less in material synthesis itself than in the reproducible integration of MoS_2_ SAs into fiber laser cavities with commercially viable yield rates. Solution-based deposition techniques such as inkjet printing present promising pathways for scalable fabrication, as demonstrated for various 2D materials [9]; however, they demand precise regulation of solvent evaporation kinetics and film uniformity to minimize performance variation over large-area substrates, and MoS_2_-specific process optimization remains limited. While recent progress in in situ polymerization and direct growth on fiber end-faces has alleviated certain integration challenges, comprehensive process windows tailored for industrial-scale manufacturing have yet to be established.

Long-term reliability exceeding 1000 h constitutes one of the most demanding criteria for industrial qualification, and existing literature offers limited empirical evidence to confirm that MoS_2_ SAs can consistently satisfy this benchmark. Short-term stability evaluations (typically <100 h), indicate sufficient robustness under moderate intracavity intensities [192], but accelerated aging data under realistic thermal and optical stress conditions remain sparse. Potential degradation pathways, inferred from general 2D material studies, may include photo-oxidation at edge sites, interfacial delamination driven by cyclic thermal loading, and defect-mediated fatigue of absorption saturation, though direct mechanistic evidence for MoS_2_ SAs is still lacking. Encapsulation strategies employing hexagonal boron nitride capping layers have shown promise in enhancing material stability under harsh conditions [193]; however, their capacity to sustain device performance during continuous >1000 h operation at high peak power densities lacks systematic validation. Attaining industrial-grade reliability will thus necessitate not only advances in passivation methodologies but also the development of standardized testing protocols that capture the synergistic effects of optical, thermal, and environmental stressors, drawing upon established multi-stress reliability frameworks for optoelectronic devices [194].

## Figures and Tables

**Table 1:** Key Performance Parameters of Representative Saturable Absorbers for Mode-Locked Fiber Lasers.

SA Material	Modulation Depth (%)	Response Time	Damage Threshold	Wavelength Range (μm)	Refs.
MoS_2_ (few-layer)	3.0–20.0	−30 fs	≈1.7 GW/cm^2^	1.0–2.0	[9,19,20,21,22]
Graphene	6.2–66.5	0.1–0.5 ps	—	0.8–2.5	[23,24]
CNTs	1.0–4.5	<1 ps	+130%	1.0–2.0	[25,26]
BP	−15.0	24 ± 2 fs	0.2–0.4 mJ/cm^2^	1.0–3.0	[27,28,29]
MXene (e.g., Ti_3_C_2_T_x_)	11.3–50.0	N/A	1.5–3.0 GW/cm^2^	1.0–2.5	[30]
Topological Insulator (e.g., Bi_2_Se_3_)	39.8–98.0	<1 ps	—	1.0–2.0	[31,32]
SESAM	0.39–11.5	18–100 ps	0.5–5.0 GW/cm^2^	0.8–2.0	[3,33,34]

General notes: Modulation depth is defined as (T_unsat_ − T_sat_)/T_unsat_; negative values indicate anomalous saturation (e.g., BP). Damage thresholds are reported for femtosecond pulses (typically 100–500 fs). All values represent typical experimental ranges from recent literature.

**Table 2:** Comparison of Preparation Methods for MoS_2_ SAs.

Preparation Method	Typical Layer Number	Advantages	Disadvantages	Key Performance Parameters	Refs.
Mechanical Exfoliation (Scotch Tape Method)	Monolayer/ Few-layer	High crystal quality Low defect density Simple operation	Small flake size Low yield Poor controllability	Modulation Depth: ~2–5% Non-saturable Loss: Low	[10,36]
Chemical Vapor Deposition	Monolayer/ Controlled layers	Large area growth Uniform thickness control High optical quality	High equipment cost Complex transfer process High temperature required	Modulation Depth: 5–10% Damage Threshold: High	[54,67]
Liquid Phase Exfoliation	Few-layer/ Multi-layer	Low cost Scalable production Easy integration with polymers	Lateral size distribution Potential solvent residues Restacking issues	Modulation Depth: 3–8% Saturation Intensity: Moderate	[56,57]
Hydrothermal Method	Multi-layer/ Nanoflowers	Environmentally friendly Good crystallinity Controlled morphology	Long reaction time Aggregation tendency	Modulation Depth: >10% Stability: Good	[44]
Fiber Integration/Deposition	—	All-fiber compatibility Low insertion loss Compact structure	Fragile structure Limited interaction length	Modulation Depth: <5% Insertion Loss: Very Low	[12,42]

**Table 3:** Performance Comparison of Different MoS_2_-Based Composite SAs.

Composite Structure Type	Modulation Depth (%)	Saturation Intensity (MW/cm^2^)	Damage Threshold (GW/cm^2^)	Response Time (ps/fs)	Operating Band (nm)	Measurement Conditions (Wavelength/Pulse Width)	References
MoS_2_/Graphene Heterostructure	5.8–6.5	15–25	>1.5	<500 fs	1000/1550	Z-scan @ 800 nm, ~140 fs; Mode-locking @ 1064 nm, ~350 fs	[96,127]
MoS_2_/BP	4.2–5.0	10–18	0.3–0.5	<300 fs	2000	Q-switching @ 1094 nm, ~100 ns; Mode-locking @ 2000 nm, ps regime	[24,120,121,122,123,124,125,126,127,128]
MoS_2_/Polymer	2.5–3.8	20–40	>1.2	0.5–1.0 ps	1000/1550	Mode-locking @ 1550 nm, ~500 fs	[74]
MoS_2_/Metal Plasmonic	>8.0	<10	0.2–0.4	<200 fs	1550	Z-scan @ 800 nm, ~100 fs; Mode-locking @ 1550 nm, ~200 fs	[131,132]
Mo_0.5_W_0.5_S_2_ Alloy	3.5–4.5	20–40	>1.2	0.5–1.0 ps	1000/1550	Q-switching @ 1550 nm, ~800 fs	[133]

General notes: Z-scan, open-aperture Z-scan technique; Q-switching, passively Q-switched operation; Mode-locking, passively mode-locked operation.

## Data Availability

No new data were created or analyzed in this study. Data sharing is not applicable to this article.

## References

[1] Woodward R.I., Kelleher E.J.R. (2015). 2D Saturable Absorbers for Fibre Lasers. Appl. Sci..

[2] Keller U. (2003). Recent Developments in Compact Ultrafast Lasers. Nature.

[3] Keller U., Weingarten K.J., Kärtner F.X., Kopf D., Braun B., Jung I.D., Fluck R., Höninger C., Matuschek N., Aus der Au J. (1996). Semiconductor Saturable Absorber Mirrors (SESAM’s) for Femtosecond to Nanosecond Pulse Generation in Solid-State Lasers. IEEE J. Sel. Top. Quantum Electron..

[4] Zhang M., Wu Q., Zhang F., Chen L., Jin X., Hu Y., Zheng Z., Zhang H. (2019). 2D Black Phosphorus Saturable Absorbers for Ultrafast Photonics. Adv. Opt. Mater..

[5] Debnath P.C., Yeom D.-I. (2021). Ultrafast Fiber Lasers with Low-Dimensional Saturable Absorbers: Status and Prospects. Sensors.

[6] Krasnok A., Lepeshov S., Alù A. (2018). Nanophotonics with 2D Transition Metal Dichalcogenides. Opt. Express.

[7] Wang G.H., Baker-Murray A.A., Blau W.J. (2019). Saturable Absorption in 2D Nanomaterials and Related Photonic Devices. Laser Photonics Rev..

[8] Liu H., Luo A.-P., Wang F.-Z., Tang R., Liu M., Luo Z.-C., Xu W.-C., Zhao C.-J., Zhang H. (2014). Femtosecond Pulse Erbium-Doped Fiber Laser by a Few-Layer MoS_2_ Saturable Absorber. Opt. Lett..

[9] Liu M., Zheng X.W., Qi Y.L., Liu H., Luo A.P., Luo Z.C., Xu W.C., Zhao C.J., Zhang H. (2014). Microfiber-Based Few-Layer MoS_2_ Saturable Absorber for 2.5 GHz Passively Harmonic Mode-Locked Fiber Laser. Opt. Express.

[10] Ma P.F., Lin W., Zhang H.N., Xu S.H., Yang Z.M. (2019). High-Power Large-Energy Rectangular Mode-Locked Er-Doped Fiber Laser Based on High-Damage-Threshold MoS_2_ Saturable Absorber. IEEE Photon. J..

[11] Woodward R.I., Howe R.C.T., Hu G., Torrisi F., Zhang M., Hasan T., Kelleher E.J.R. (2015). Few-Layer MoS_2_ Saturable Absorbers for Short-Pulse Laser Technology: Current Status and Future Perspectives [Invited]. Photonics Res..

[12] Ma C., Wang C., Gao B., Adams J., Wu G., Zhang H. (2019). Recent Progress in Ultrafast Lasers Based on 2D Materials as a Saturable Absorber. Appl. Phys. Rev..

[13] Jiang T., Yin K., Wang C., You J., Ouyang H., Miao R., Zhang C., Wei K., Li H., Chen H. (2020). Ultrafast Fiber Lasers Mode-Locked by Two-Dimensional Materials: Review and Prospect. Photonics Res..

[14] Zhang H.N., Sun S., Shang X.X., Guo B., Li X.H., Chen X.H., Jiang S.Z., Zhang H., Ågren H., Zhang W.F. (2022). Ultrafast Photonics Applications of Emerging 2D-Xenes Beyond Graphene. Nanophotonics.

[15] Wang J., Wang X., Lei J., Ma M., Wang C., Ge Y., Wei Z. (2020). Recent Advances in Mode-Locked Fiber Lasers Based on Two-Dimensional Materials. Nanophotonics.

[16] Zhang B., Liu J., Wang C., Yang K., Lee C., Zhang H., He J. (2020). Recent Progress in 2D Material-Based Saturable Absorbers for All Solid-State Pulsed Bulk Lasers. Laser Photon. Rev..

[17] Wang Y., Wang J., Wen Q. (2021). MXene/Graphene Oxide Heterojunction as a Saturable Absorber for Passively Q-Switched Solid-State Pulse Lasers. Nanomaterials.

[18] Chen S., Wang F., Kuang F., Kang S., Liang H., Zheng L., Guan L., Wu Q. (2022). Femtosecond Pulsed Fiber Laser by an Optical Device Based on NaOH-LPE Prepared WSe_2_ Saturable Absorber. Nanomaterials.

[19] Huang Y., Luo Z., Li Y., Zhong M., Xu B., Che K., Xu H., Cai Z., Peng J., Weng J. (2014). Widely-tunable, passively Q-switched erbium-doped fiber laser with few-layer MoS_2_ saturable absorber. Opt. Express.

[20] Wang K.P., Wang J., Fan J.T., Lotya M., O’Neill A., Fox D., Feng Y.Y., Zhang X.Y., Jiang B.X., Zhao Q.Z. (2013). Ultrafast Saturable Absorption of Two-Dimensional MoS_2_ Nanosheets. ACS Nano.

[21] Wang S., Yu H., Zhang H., Wang A., Zhao M., Chen Y., Mei L., Wang J. (2014). Broadband Few-Layer MoS_2_ Saturable Absorbers. Adv. Mater..

[22] Li Y., Dong N., Zhang S., Zhang X., Feng Y., Wang K., Zhang L., Wang J. (2015). Giant two-photon absorption in monolayer MoS_2_. Laser Photonics Rev..

[23] Sun Z., Hasan T., Torrisi F., Popa D., Privitera G., Wang F., Bonaccorso F., Basko D.M., Ferrari A.C. (2010). Graphene Mode-Locked Ultrafast Laser. ACS Nano.

[24] Bao Q.L., Zhang H., Wang Y., Ni Z.H., Yan Y.L., Shen Z.X., Loh K.P., Tang D.Y. (2009). Atomic-Layer Graphene as a Saturable Absorber for Ultrafast Pulsed Lasers. Adv. Funct. Mater..

[25] Kivisto S., Hakulinen T., Kaskela A., Aitchison B., Brown D.P., Nasibulin A.G., Kauppinen E.I., Härkönen A., Okhotnikov O.G. (2009). Carbon nanotube films for ultrafast broadband technology. Opt. Express.

[26] Yamashita S. (2012). A tutorial on nonlinear photonic applications of carbon nanotube and graphene. J. Light. Technol..

[27] Chen Y., Jiang G.B., Chen S.Q., Guo Z.N., Yu X.F., Zhao C.J., Zhang H., Bao Q.L., Wen S.C., Tang D.Y. (2015). Mechanically Exfoliated Black Phosphorus as a New Saturable Absorber for Both Q-Switching and Mode-Locking Laser Operation. Opt. Express.

[28] Luo Z.C., Liu M., Guo Z.N., Jiang X.F., Luo A.P., Zhao C.J., Yu X.F., Xu W.C., Zhang H. (2015). Microfiber-Based Few-Layer Black Phosphorus Saturable Absorber for Ultra-Fast Fiber Laser. Opt. Express.

[29] Jiang T., Yin K., Zheng X., Yu H., Cheng X.A. (2015). Black phosphorus as a new broadband saturable absorber for infrared passively Q-switched fiber lasers. arXiv.

[30] Feng J., Li X., Feng T., Wang Y., Liu J., Zhang H. (2020). Harmonic mode-locked Er-doped fiber laser by evanescent field-based MXene Ti_3_C_2_T_x_ (T = F, O, or OH) saturable absorber. Ann. Phys..

[31] Yan P.G., Lin R., Ruan S.C., Liu A.J., Chen H., Zheng Y.Q., Chen S.F., Guo C.Y., Hu J.G. (2015). A Practical Topological Insulator Saturable Absorber for Mode-Locked Fiber Laser. Sci. Rep..

[32] Wei R., Zhang H., Tian X., Qiao T., Hu Z., Chen Z., He X., Yu Y., Qiu J. (2016). MoS_2_ nanoflowers as high performance saturable absorbers for an all-fiber passively Q-switched erbium-doped fiber laser. Nanoscale.

[33] Finke T., Nürnberg J., Sichkovskyi V., Golling M., Keller U., Reithmaier J.P. (2020). Temperature resistant fast In_x_Ga_1-x_As/GaAs quantum dot saturable absorber for epitaxial integration into semiconductor surface emitting lasers. Opt. Express.

[34] Saraceno C.J., Heckl O.H., Baer C.R.E., Schriber C., Golling M., Beil K., Kränkel C., Südmeyer T., Huber G., Keller U. (2012). Sub-100 femtosecond pulses from a SESAM mode-locked thin disk laser. Appl. Phys. B.

[35] Mirershadi S., Sattari F., Alipour A., Mortazavi S.Z. (2020). Non-linear thermo-optical properties of MoS_2_ nanoflakes by means of the Z-scan technique. Front. Phys..

[36] Aiub E.J., Steinberg D., de Souza E.A.T., Saito L.A.M. (2017). 200-fs mode-locked Erbium-doped fiber laser by using mechanically exfoliated MoS_2_ saturable absorber onto D-shaped optical fiber. Opt. Express.

[37] Sotor J., Sobon G., Macherzynski W., Paletko P., Grodecki K., Abramski K.M. (2014). Mode-locking in Er-doped fiber laser based on mechanically exfoliated Sb_2_Te_3_ saturable absorber. Opt. Mater. Express.

[38] Niu K.D., Sun R.Y., Chen Q.Y., Man B.Y., Zhang H.N. (2018). Passively mode-locked Er-doped fiber laser based on SnS_2_ nanosheets as a saturable absorber. Photon. Res..

[39] Lei J., Wang J., Wang X., Wei Z. (2022). Ternary 2D Mo(_1−x)_W_x_S_2_ as a saturable absorber for femtosecond mode-locked all fiber lasers. Opt. Laser Technol..

[40] Wang Y., Wang Y., Dong Y., Zhou L., Kang J., Wang N., Li Y., Yuan X., Zhang Z., Huang H. (2023). 2D Nb_2_CT_x_ MXene/MoS_2_ Heterostructure Construction for Nonlinear Optical Absorption Modulation. Opto-Electron. Adv..

[41] Luo Z., Huang Y., Zhong M., Li Y., Wu J., Xu B., Xu H., Cai Z., Peng J., Weng J. (2014). 1-, 1.5-, and 2-μm Fiber Lasers Q-Switched by a Broadband Few-Layer MoS_2_ Saturable Absorber. J. Light. Technol..

[42] Liu C., Xu Q., Li Z., He J., Wang P., Huang Y., Liu Y., Wang Z. (2022). Ti_3_C_2_T_x_ as Saturable Absorber for Highly Stable All-Fiber Er-Doped Q-Switched Laser. IEEE Photonics Technol. Lett..

[43] Xu B., Cheng Y.J., Wang Y., Huang Y.Z., Peng J., Luo Z.Q., Xu H.Y., Cai Z.P., Weng J., Moncorgé R. (2014). Passively Q-switched Nd:YAlO_3_ nanosecond laser using MoS_2_ as saturable absorber. Opt. Express.

[44] Huang B., Zheng M., Zhao Y., Wu J., Thong J.T. (2019). Atomic Layer Deposition of High-Quality Al_2_O_3_ Thin Films on MoS_2_ with Water Plasma Treatment. ACS Appl. Mater. Interfaces.

[45] Lv R., Chen Z., Liu S., Wang J., Li Y., Wang Y., Wang Y. (2019). Optical properties and applications of molybdenum disulfide/SiO_2_ saturable absorber fabricated by sol-gel technique. Opt. Express.

[46] Splendiani A., Sun L., Zhang Y., Li T., Kim J., Chim C.Y., Galli G., Wang F. (2010). Emerging Photoluminescence in Monolayer MoS_2_. Nano Lett..

[47] Du J., Wang Q.K., Jiang G.B., Xu C.W., Zhao C.J., Xiang Y.J., Chen Y., Wen S.C., Zhang H. (2014). Ytterbium-doped fiber laser passively mode locked by few-layer Molybdenum Disulfide (MoS_2_) saturable absorber functioned with evanescent field interaction. Sci. Rep..

[48] Yu Z., Ong Z.-Y., Pan Y., Cui Y., Xin R., Shi Y., Wang B., Wu Y., Chen T., Zhang Y.-W. (2016). Realization of Room-Temperature Phonon-Limited Carrier Transport in Monolayer MoS_2_ by Dielectric and Carrier Screening. Adv. Mater..

[49] Adilbekova B., Lin Y., Yengel E., Faber H., Harrison G., Firdaus Y., El-Laban A., Anjum D.H., Tung V., Anthopoulos T.D. (2020). Liquid phase exfoliation of MoS_2_ and WS_2_ in aqueous ammonia and their application in highly efficient organic solar cells. J. Mater. Chem. C.

[50] Li X., Du L.J., Lu X.B., Yang R., Shi D.X., Zhang G.Y. (2017). A facile and efficient dry transfer technique for two-dimensional van der Waals heterostructures. Chin. Phys. B.

[51] Aryeetey F., Pourianejad S., Ayanbajo O., Nowlin K., Ignatova T., Aravamudhan S. (2021). Bandgap Recovery of Monolayer MoS_2_ Using Defect Engineering and Chemical Doping. RSC Adv..

[52] Xia H.D., Li H.P., Lan C.Y., Li C., Zhang X.X., Zhang S.J., Liu Y. (2014). Ultrafast erbium-doped fiber laser mode-locked by a CVD-grown molybdenum disulfide (MoS_2_) saturable absorber. Opt. Express.

[53] Yu H., Liao M., Zhao W., Liu G., Xu X., Wei Z., Xu K., Liu K., Hu Z., Deng K. (2017). Wafer-scale growth and transfer of highly-oriented monolayer MoS_2_ continuous films. ACS Nano.

[54] Liu M.-L., OuYang Y.-Y., Hou H.-R., Lei M., Liu W.-J., Wei Z.-Y. (2018). MoS_2_ saturable absorber prepared by chemical vapor deposition method for nonlinear control in Q-switching fiber laser. Chin. Phys. B.

[55] Yan L., Tang X., Huang L., Chen B. (2021). Adaptive mask generating algorithm based on the fuzzy set theory for the weighted least-squares phase unwrapping. Opt. Lasers Eng..

[56] Fu B., Sun J., Wang G., Shang C., Ma Y., Ma J., Xu L., Scardaci V. (2020). Solution-processed two-dimensional materials for ultrafast fiber lasers(invited). Nanophotonics.

[57] Coleman J.N., Lotya M., O’Neill A., Bergin S.D., King P.J., Khan U., Young K., Gaucher A., De S., Smith R.J. (2011). Two-Dimensional Nanosheets Produced by Liquid Exfoliation of Layered Materials. Science.

[58] Kong L.C., Xie G.Q., Yuan P., Qin Z.P., Guo Z., He H., Zhao L.M., Tang D.Y. (2015). Passive Q-switching and Q-switched mode-locking operations of 2 μm Tm:CLNGG laser with MoS_2_ saturable absorber mirror. Photonics Res..

[59] Wang C., Chen T., Meng Z., Niu S., Li Z., Yang X. (2024). Mo_2_TiAlC_2_ as a saturable absorber for a passively Q-switched Tm:YAlO3 laser. Nanomaterials.

[60] Zhang Y., Wu Z., Sun J., Sun Q., Chen F., Zhang M., Duan H. (2023). Synthesis and sensing performance of chitin fiber/MoS_2_ composites. Nanomaterials.

[61] Kaur S., Pandey R., Karna S.P. (2021). Enhanced nonlinear optical response of graphene-based nanoflake van der Waals heterostructures. RSC Adv..

[62] Wang Z.T., Xu Y.H., Dhanabalan S.C., Sophia J., Zhao C.J., Xu C.W., Xiang Y.J., Li J.Q., Zhang H. (2016). Black phosphorus quantum dots as an efficient saturable absorber for bound soliton operation in an erbium doped fiber laser. IEEE Photonics J..

[63] Liu M., Liu W., Wei Z. (2019). MoTe_2_ Saturable Absorber With High Modulation Depth for Erbium-Doped Fiber Laser. J. Light. Technol..

[64] Zhan Y.J., Liu Z., Najmaei S., Ajayan P.M., Lou J. (2012). Large-area vapor-phase growth and characterization of MoS_2_ atomic layers on a SiO_2_ substrate. Small.

[65] Zhou G., Wang J., Cheng W., Nan H., Zhao X., Wei H., Xue C., Ma Y., Yang P. (2024). Surface modified molybdenum disulfide nanosheets for corrosion resistance improvement on polyurethane coatings. Corros. Rev..

[66] Tao L., Chen K., Chen Z., Chen W., Gui X., Chen H., Li X., Xu J.-B. (2017). Centimeter-scale CVD growth of highly crystalline single-layer MoS_2_ film with spatial homogeneity and the visualization of grain boundaries. ACS Appl. Mater. Interfaces.

[67] Ng E.K., Lau K.Y., Lee H.K., Yusoff N.M., Sarmani A.R., Omar M.F., Mahdi M.A. (2021). L-Band Femtosecond Fiber Laser Based on a Reduced Graphene Oxide Polymer Composite Saturable Absorber. Opt. Mater. Express.

[68] Wu Z., Ni Z. (2016). Spectroscopic Investigation of Defects in Two-Dimensional Materials. Nanophotonics.

[69] Lee C., Yan H., Brus L.E., Heinz T.F., Hone J., Ryu S. (2010). Anomalous lattice vibrations of single- and few-layer MoS_2_. ACS Nano.

[70] Mao D., Wang Y., Ma C., Han L., Jiang B., Gan X., Hua S., Zhang W., Mei T., Zhao J. (2015). WS_2_ mode-locked ultrafast fiber laser. Sci. Rep..

[71] Sathiyan S., Velmurugan V., Senthilnathan K., Ramesh Babu P., Sivabalan S. (2016). All-normal dispersion passively mode-locked Yb-doped fiber laser using MoS_2_–PVA saturable absorber. Laser Phys..

[72] Liu W., Pang L., Han H., Liu M., Lei M., Fang S., Teng H., Wei Z. (2017). Tungsten Disulfide Saturable Absorbers for 67 fs Mode-Locked Erbium-Doped Fiber Lasers. Opt. Express.

[73] Wang Q., Ge S., Li X., Qiu J., Ji Y., Feng J., Sun D. (2013). Valley carrier dynamics in monolayer molybdenum disulfide from helicity-resolved ultrafast pump-probe spectroscopy. ACS Nano.

[74] Ahmed M., Latiff A., Arof H., Ahmad H., Harun S. (2016). Femtosecond mode-locked erbium-doped fiber laser based on MoS_2_-PVA saturable absorber. Opt. Laser Technol..

[75] Zhang Y., Zhu J.Q., Li P.X., Wang X., Yu H., Xiao K., Li C., Zhang G. (2018). All-Fiber Yb-Doped Fiber Laser Passively Mode-Locking by Monolayer MoS_2_ Saturable Absorber. Opt. Commun..

[76] Liu W.-J., Liu M., OuYang Y., Hou H., Ma G., Lei M., Wei Z. (2018). Tungsten diselenide for mode-locked erbium-doped fiber lasers with short pulse duration. Nanotechnology.

[77] Zhang Y., Liu W., Li Z., Cheng H., Zhang Y., Jia G., Chen S., Tian J. (2017). Ultrathin polarization-insensitive wide-angle broadband near-perfect absorber in the visible regime based on few-layer MoS_2_ films. Appl. Phys. Lett..

[78] Lü R., Wang Y., Wang J., Ren W., Li L., Liu S., Chen Z., Li Y., Wang H., Fu F. (2019). Soliton and bound-state soliton mode-locked fiber laser based on a MoS_2_/fluorine mica Langmuir–Blodgett film saturable absorber. Photonics Res..

[79] Liu H., Zheng X.W., Liu M., Zhao N., Luo A.P., Luo Z.C., Xu W.C., Zhang H., Zhao C.J., Wen S.-C. (2014). Femtosecond pulse generation from a topological insulator mode-locked fiber laser. Opt. Express.

[80] Zapata J.D., Steinberg L., Saito L.A.M., de Oliveira R.E.P., Cardenas A.M., Thoroh de Souza E.A. (2016). Efficient graphene saturable absorbers on D-shaped optical fiber for ultrashort pulse generation. Sci. Rep..

[81] Bao X.Z., Mu H.K., Chen Y., Li P.F., Li L., Li S.J., Qasim K., Zhang Y.P., Zhang H., Bao Q.L. (2018). Ytterbium-doped fiber laser passively mode locked by evanescent field interaction with CH3NH3SnI3 perovskite saturable absorber. J. Phys. D Appl. Phys..

[82] Li H., Tan Y., Liu P., Guo C., Luo M., Han J., Lin T., Huang F., Chen M., Cao G. (2016). Atomic-Sized Pores Enhanced Electrocatalysis of TaS_2_ Nanosheets for Hydrogen Evolution. Adv. Mater..

[83] Lu S., Zhao C., Zou Y., Chen S., Chen Y., Li Y., Zhang H., Wen S., Tang D. (2013). Third-order nonlinear optical properties of topological insulator Bi_2_Se_3_. Opt. Express.

[84] Kelley P.L. (1965). Self-Focusing of Optical Beams. Phys. Rev. Lett..

[85] Wang Z., He R., Liu Y.-g., Zhang H., Han S., Li H., Wang G., Yang G., Wang Z. (2018). Generation of trapezoidal envelope pulses and soliton rains from passively mode-locked fiber laser with MoS_2_ saturable absorber on microfiber. Appl. Phys. Express.

[86] Tang D.Y., Zhao L.M., Zhao B., Liu A.Q. (2005). Mechanism of Multi-Soliton Formation and Soliton Energy Quantization in Passively Mode-Locked Fiber Lasers. Phys. Rev. A.

[87] Zhang H., Tang D.Y., Zhao L.M., Knize R.J. (2010). Vector Dark Domain Wall Solitons in a Fiber Ring Laser. Opt. Express.

[88] Wang F. (2017). Two-dimensional materials for ultrafast lasers. Chin. Phys. B.

[89] Zhang H., Bao Q., Tang D., Zhao L., Loh K. (2009). Large-Energy Soliton Erbium-Doped Fiber Laser with a Graphene-Polymer Composite Mode-Locker. Appl. Phys. Lett..

[90] Song Y.F., Zhang H., Tang D.Y., Shen D.Y. (2012). Polarization Rotation Vector Solitons in a Graphene Mode-Locked Fiber Laser. Opt. Express.

[91] Wang Y., Mao D., Gan X., Han L., Ma C., Xi T., Zhang Y., Shang W., Hua S., Zhao J. (2015). Harmonic mode locking of bound-state solitons fiber laser based on MoS_2_ saturable absorber. Opt. Express.

[92] Li L., Lv R., Chen Z., Wang J., Liu S., Ren W., Wang Y. (2019). Mode-Locked Er-Doped Fiber Laser by Using MoS_2_/SiO_2_ Saturable Absorber. Nanoscale Res. Lett..

[93] Woodward R.I., Kelleher E.J.R., Howe R.C.T., Hu G., Torrisi F., Hasan T., Popov S.V., Taylor J.R. (2014). Tunable Q-switched Fiber Laser Based on Saturable Edge-State Absorption in Few-Layer Molybdenum Disulfide (MoS_2_). Opt. Express.

[94] Peng B., Yu G., Liu X., Liu B., Liang X., Bi L., Deng L., Sum T.C., Loh K.P. (2016). Ultrafast charge transfer in MoS_2_/WSe_2_ p-n heterojunction. 2D Mater..

[95] Ke D., Sui L.Z., Liu D.L., Cui J.Q., Zhang Y.F., Li Q.Y., Li S.Y., Jiang Y.F., Chen A.M., Song J.L. (2018). Ultrafast Dynamics of Defect-Assisted Carrier Capture in MoS_2_ Nanodots Investigated by Transient Absorption Spectroscopy. Chin. J. Chem. Phys..

[96] Sun X.D., Zhang B., Li Y., Luo X.Q., Li G.L., Chen Y., Zhang C.H., He J. (2018). Tunable Ultrafast Nonlinear Optical Properties of Graphene/MoS_2_ fVan der Waals Heterostructures and Their Application in Solid-State Bulk Lasers. ACS Nano.

[97] Kistner-Morris J., Shi A., Liu E., Arp T., Farahmand F., Taniguchi T., Watanabe K., Aji V., Lui C.H., Gabor N. (2024). Electric-field tunable Type-I to Type-II band alignment transition in MoSe_2_/WS_2_ heterobilayers. Nat. Commun..

[98] Yang X.L., Chen Y., Zhao C.J., Zhang H. (2014). Pulse dynamics controlled by saturable absorber in a dispersion-managed normal dispersion Tm-doped mode-locked fiber laser. Chin. Opt. Lett..

[99] Sun Z., Lin X.C., Popa D., Yu H.J., Hasan T., Torrisi F., Kelleher E.J.R., Zhang L., Sun L., Guo L. Wideband tunable, high-power, graphene mode-locked ultrafast lasers. Proceedings of the 2011 Conference on Lasers and Electro-Optics Europe and 12th European Quantum Electronics Conference (CLEO EUROPE/EQEC).

[100] Ahmad H., Ismail M.A., Suthaskumar M., Tiu Z.C., Harun S.W., Zulkifli M.Z., Samikannu S., Sivaraj S. (2016). S-band Q-switched fiber laser using molybdenum disulfide (MoS_2_) saturable absorber. Laser Phys. Lett..

[101] Nie Z., Long R., Teguh J.S., Huang C.C., Hewak D.W., Yeow E.K.L., Shen Z., Prezhdo O.V., Loh Z.H. (2015). Ultrafast electron and hole relaxation pathways in few-layer MoS_2_. J. Phys. Chem. C.

[102] Ahmed M.H.M., Latiff A.A., Arof H., Harun S.W. (2016). Mode-locking pulse generation with MoS_2_–PVA saturable absorber in both anomalous and ultra-long normal dispersion regimes. Appl. Opt..

[103] Khazaeizhad R., Kassani S.H., Jeong H., Yeom D.I., Oh K. (2014). Mode-locking of Er-doped fiber laser using a multilayer MoS_2_ thin film as a saturable absorber in both anomalous and normal dispersion regimes. Opt. Express.

[104] Ling W., Xu H., Yang J., Qiu X., He T., An P., Bi C., Yuan S., Wang M., Tian X. (2024). 1.4 W Passively Q-Switched Mode-Locked Tm:CALGO Laser with a MoS_2_ Saturable Absorber. Photonics.

[105] Cui Y., Lu F., Liu X. (2016). MoS_2_-clad microfibre laser delivering conventional, dispersion-managed and dissipative solitons. Sci. Rep..

[106] Zhang B., Xu Z., Ning Z., Bai X., Zheng J., Zhang Q., Mi S., Sun W., Kauppinen E.I., Li L. (2025). Fabrication of a CVD-grown MoS_2_/DWCNT heterostructure as a saturable absorber for a mode-locked fiber laser. Chaos Solitons Fractals.

[107] Villanueva G.E., Pérez-Millán P. (2012). Dynamic control of the operation regimes of a mode-locked fiber laser based on intracavity polarizing fibers: Experimental and theoretical validation. Opt. Lett..

[108] Zhang R., Wang J., Zhang X.Y., Lin J.T., Li X., Kuan P.W., Zhou Y., Liao M.S., Gao W.Q. (2019). Mode-Locked Fiber Laser with MoSe_2_ Saturable Absorber Based on Evanescent Field. Chin. Phys. B.

[109] Ma P., Lin W., Zhang H., Xu S., Yang Z. (2019). High-Power Large-Energy Raman Soliton Generations Within a Mode-Locked Yb-Doped Fiber Laser Based on High-Damage-Threshold CVD-MoS_2_ as Modulator. Nanomaterials.

[110] Zhang A., Wang Z., Ouyang H., Lyu W., Sun J., Cheng Y., Fu B. (2021). Recent Progress of Two-Dimensional Materials for Ultrafast Photonics. Nanomaterials.

[111] Zhou L., Liu Y., Xie G.H., Zhang W., Zhu Z., Ouyang C., Gu C., Li W. (2019). Generation of stretched pulses from an all-polarization-maintaining Er-doped mode-locked fiber laser using nonlinear polarization evolution. Appl. Phys. Express.

[112] Chen B., Zhang X., Wu K., Wang H., Wang J., Chen J. (2015). Q-Switched Fiber Laser Based on Transition Metal Dichalcogenides MoS_2_, MoSe_2_, WS_2_, and WSe_2_. Opt. Express.

[113] Zhang H., Ma P., Zhu M., Zhang W., Wang G., Fu S. (2020). Palladium Selenide as a Broadband Saturable Absorber for Ultra-Fast Photonics. Nanophotonics.

[114] Zhang S., Dong N., McEvoy N., O’Brien M., Winters S., Berner N.C., Yim C., Li Y., Zhang X., Chen Z. (2015). Direct Observation of Degenerate Two-Photon Absorption and Its Saturation in WS_2_ and MoS_2_ Monolayer and Few-Layer Films. ACS Nano.

[115] Xi Q., Yang J., Xie J., Wang X., Xu F., Li Q., Zhang K., Wang T., Su F. (2025). Terahertz Saturable Absorption across Charge Separation in Photoexcited Monolayer Graphene/MoS_2_ Heterostructure. J. Phys. Chem. Lett..

[116] Chen H., Yin J., Yang J., Zhang X., Liu M., Jiang Z., Wang J., Sun Z., Guo T., Liu W. (2017). Transition-Metal Dichalcogenides Heterostructure Saturable Absorbers for Ultrafast Photonics. Opt. Lett..

[117] Xie L.M. (2015). Two-dimensional transition metal dichalcogenide alloys: Preparation, characterization and applications. Nanoscale.

[118] Zhang C., Gong C., Nie Y., Min K.-A., Liang C., Oh Y.J., Zhang H., Wang W., Hong S., Colombo L. (2017). Systematic study of electronic structure and band alignment of monolayer transition metal dichalcogenides in van der Waals heterostructures. 2D Mater..

[119] Singh D.K., Vaidya A., Thomas V., Theodore M., Kore S., Vaidya U. (2020). Finite element modeling of the fiber-matrix interface in polymer composites. J. Compos. Sci..

[120] Xue Y., Xie Z., Ye Z., Hu X., Xu J., Zhang H. (2018). Enhanced saturable absorption of MoS_2_-black phosphorus composite in 2 μm passively Q-switched Tm:YAP laser. Chin. Opt. Lett..

[121] Nie Z., Wang Y., Li Z., Sun Y., Qin S., Liu X., Turcu I.C.E., Shi Y., Zhang R., Ye Y. (2019). Ultrafast free carrier dynamics in black phosphorus–molybdenum disulfide (BP/MoS_2_) heterostructures. Nanoscale Horiz..

[122] Shao J., Yao G., Wu X., Lin K., Zhang S., Cheng X., Zhong D., Liu C., Liu C., Wang F. (2025). Robust Mode-Locking in All-Fiber Ultrafast Laser by Nanocavity of Two-Dimensional Heterostructure. Light Sci. Appl..

[123] Wu K., Zhang X., Wang J., Chen J. (2015). 463-MHz fundamental mode-locked fiber laser based on few-layer MoS_2_ saturable absorber. Opt. Lett..

[124] Song Y.W., Jang S.Y., Han W.S., Bae M.K. (2010). Graphene mode-lockers for fiber lasers functioned with evanescent field interaction. Appl. Phys. Lett..

[125] Halim S.N.M., Ahmad F., Lokman M.Q., Sapingi H.H.J., Taib M.F.M., Nawawi W.M.F.W., Yahaya H., Rahman M.A.A., Shafie S., Harun S.W. (2022). First principles study and experimental investigation of graphene molybdenum disulphide nanocomposites based passive saturable absorber. Photonics.

[126] Zhao C.J., Zhang H., Qi X., Chen Y., Wang Z.T., Wen S.C., Tang D.Y. (2012). Ultra-Short Pulse Generation by a Topological Insulator Based Saturable Absorber. Appl. Phys. Lett..

[127] Liu H.H., Li Z.L., Song W., Yu Y., Pang F.F., Wang T.Y. (2020). MoS_2_/Graphene Heterostructure Incorporated Passively Mode-Locked Fiber Laser: From Anomalous to Normal Average Dispersion. Opt. Mater. Express.

[128] Zhou B., Jiang K., Shang L., Zhang J., Li Y., Zhu L., Gong S.J., Hu Z., Chu J. (2020). Enhanced Carrier Separation in Ferroelectric In_2_Se_3_/MoS_2_ van der Waals Heterostructure. J. Mater. Chem. C.

[129] Ji Z., Hong H., Zhang J., Zhang Q., Huang W., Cao T., Qiao R., Liu C., Liang J., Jin C. (2017). Robust stacking-independent ultrafast charge transfer in MoS_2_/WS_2_ bilayers. ACS Nano.

[130] Ceballos F., Bellus M.Z., Chiu H.-Y., Zhao H. (2014). Ultrafast charge separation and indirect exciton formation in a MoS_2_–MoSe_2_ van der Waals heterostructure. ACS Nano.

[131] Miao R., Shu Z., Hu Y., Tang Y., Hao H., You J., Zheng X., Cheng X., Duan H., Jiang T. (2019). Ultrafast nonlinear absorption enhancement of monolayer MoS_2_ with plasmonic Au nanoantennas. Opt. Lett..

[132] Wang J., Coillet A., Demichel O., Wang Z., Rego D., Bouhelier A., Grelu P., Cluzel B. (2020). Saturable plasmonic metasurfaces for laser mode locking. Light Sci. Appl..

[133] Wang J., Dou C., Chen L., Yan H., Meng L., Zhu J., Wei Z. (2018). High energy passively Q-switched Er-doped fiber laser based on Mo_0.5_W_0.5_S_2_ saturable absorber. Opt. Mater. Express.

[134] Li D., Xiong W., Jiang L., Xiao Z., Golgir H.R., Wang M., Huang X., Zhou Y., Lin Z., Song J. (2016). Multimodal nonlinear optical imaging of MoS_2_ and MoS_2_-based van der Waals heterostructures. ACS Nano.

[135] Park N.H., Jeong H., Choi S.Y., Kim M.H., Rotermund F., Yeom D.-I. (2015). Monolayer Graphene saturable absorbers with strongly enhanced evanescent-field interaction for ultrastiff fiber laser mode-locking. Opt. Express.

[136] Zhang X., Zhang S., Xie Y., Huang J., Wang L., Cui Y., Wang J. (2018). Tailoring the nonlinear optical performance of two-dimensional MoS_2_ nanofilms via defect engineering. Nanoscale.

[137] Wang Q.H., Kalantar-Zadeh K., Kis A., Coleman J.N., Strano M.S. (2012). Electronics and optoelectronics of two-dimensional transition metal dichalcogenides. Nat. Nanotechnol..

[138] Li J., Luo H., Zhai B., Lu R., Guo Z., Zhang H., Liu Y. (2016). Black Phosphorus: A Two-Dimensional Saturable Absorption Material for Mid-Infrared Q-Switched and Mode-Locked Fiber Lasers. Sci. Rep..

[139] Mao D., Zhang S., Wang Y., Gan X., Zhang W., Mei T., Wang Y., Wang Y., Zeng H., Zhao J. (2015). WS_2_ Saturable Absorber for Dissipative Soliton Mode Locking at 1.06 and 1.55 μm. Opt. Express.

[140] Khazaeinezhad R., Kassani S.H., Jeong H., Nazari T., Yeom D.-I., Oh K. (2015). Mode-Locked All-Fiber Lasers at Both Anomalous and Normal Dispersion Regimes Based on Spin-Coated Nano-Sheets on a Side-Polished Fiber. IEEE Photonics J..

[141] Wang S., Zhou Y., Wang Y., Yan S., Li Y., Zheng W., Deng Y., Zhu Q., Xu J., Tang Y. (2016). Digital-Wavelength Ytterbium Fiber Laser Mode-Locked with MoS_2_. Laser Phys. Lett..

[142] Dai L.L., Huang Z.N., Huang Q.Q., Zhao C., Rozhin A.G., Sergeyev S.A., Al-Araimi M.S., Mou C.B. (2021). Carbon Nanotube Mode-Locked Fiber Lasers: Recent Progress and Perspectives. Nanophotonics.

[143] Li J., Zhao Y., Chen Q., Niu K., Sun R., Zhang H. (2017). Passively Mode-Locked Ytterbium-Doped Fiber Laser Based on SnS_2_ as Saturable Absorber. IEEE Photonics J..

[144] Cunningham P.D., McCreary K.M., Hanbicki A.T., Currie M., Jonker B.T., Hayden L.M. (2016). Charge Trapping and Exciton Dynamics in Large-Area CVD Grown MoS_2_. J. Phys. Chem. C.

[145] Zhang W., Lei X., Chen W., Xu H., Wang A. (2015). Modeling of Spectral Changes in Bent Fiber Bragg Gratings. Opt. Lett..

[146] He M., Quan C., He C., Huang Y., Zhu L., Yao Z., Zhang S., Bai J., Xu X. (2017). Enhanced Nonlinear Saturable Absorption of MoS_2_/Graphene Nanocomposite Films. J. Phys. Chem. C.

[147] Woodward R.I., Howe R.C.T., Runcorn T.H., Hu G., Torrisi F., Kelleher E.J.R., Hasan T. (2015). Wideband saturable absorption in few-layer molybdenum diselenide (MoSe_2_) for Q-switching Yb-, Er- and Tm-doped fiber lasers. Opt. Express.

[148] Rusdi M.F.M., Latiff A.A., Hanafi E., Mahyuddin M.B.H., Shamsudin H., Dimyati K., Harun S.W. (2016). Molybdenum Disulphide Tape Saturable Absorber for Mode-Locked Double-Clad Ytterbium-Doped All-Fiber Laser Generation. Chin. Phys. Lett..

[149] Duan L.N., Su Y.L., Wang Y.G., Li L., Wang X., Wang Y.S. (2016). Passively Mode-Locked Erbium-Doped Fiber Laser via a D-Shape-Fiber-Based MoS_2_ Saturable Absorber with a Very Low Nonsaturable Loss. Chin. Phys. B.

[150] Dai X., Zhang X., Kislyakov I.M., Wang L., Huang J., Zhang S., Dong N., Wang J. (2019). Enhanced two-photon absorption and two-photon luminescence in monolayer MoS_2_ and WS2 by defect repairing. Opt. Express.

[151] Yu L., Chen Y., Zhang W., Yang P., Feng X. (2025). Spin-Orbit-Coupling-Governed Optical Absorption in Bilayer MoS_2_ via Strain, Twist, and Electric Field Engineering. Nanomaterials.

[152] Fang Y., Ge Y., Wang C., Zhang H. (2020). Mid-Infrared Photonics Using 2D Materials: Status and Challenges. Laser Photonics Rev..

[153] Zou X., Leng Y.X., Li Y.Y., Feng Y.Y., Zhang P.X., Hang Y., Wang J. (2015). Passively Q-switched mode-locked Tm:LLF laser with a MoS_2_ saturable absorber. Chin. Opt. Lett..

[154] Mak K.F., Lee C., Hone J., Shan J., Heinz T.F. (2010). Atomically Thin MoS_2_: A New Direct-Gap Semiconductor. Phys. Rev. Lett..

[155] Yoo C., Yoon J., Kaium M.G., Osorto B., Han S.S., Kim J.H., Kim B.K., Chung H.-S., Kim D.-J., Jung Y. (2022). Large-area vertically aligned 2D MoS_2_ layers on TEMPO-cellulose nanofibers for biodegradable transient gas sensors. Nanotechnology.

[156] Wang H.N., Zhang C.J., Rana F. (2015). Ultrafast Dynamics of Defect-Assisted Electron-Hole Recombination in Monolayer MoS_2_. Nano Lett..

[157] Zhang H., Lu S.B., Zheng J., Du J., Wen S.C., Tang D.Y., Loh K.P. (2014). Molybdenum Disulfide (MoS_2_) as a Broadband Saturable Absorber for Ultra-Fast Photonics. Opt. Express.

[158] Hasan T., Sun Z., Tan P.H., Popa D., Flahaut E., Kelleher E.J.R., Bonaccorso F., Wang F.Q., Jiang Z., Torrisi F. (2014). Double-Wall Carbon Nanotubes for Wide-Band, Ultrafast Pulse Generation. ACS Nano.

[159] Set S.Y., Yaguchi H., Tanaka Y., Jablonski M. (2004). Laser Mode Locking Using a Saturable Absorber Incorporating Carbon Nanotubes. J. Light. Technol..

[160] Xu S., Wang F.Q., Zhu C.H., Meng Y.F., Liu Y.J., Liu W.Q., Tang J.Y., Liu K.H., Hu G.H., Howe R.C.T. (2016). Ultrafast Nonlinear Photoresponse of Single-Wall Carbon Nanotubes: A Broadband Degenerate Investigation. Nanoscale.

[161] Shi H., Yan R., Bertolazzi S., Brivio J., Gao B., Kis A., Jena D., Xing H.G., Huang L. (2013). Exciton Dynamics in Suspended Monolayer and Few-Layer MoS_2_ 2D Crystals. ACS Nano.

[162] Yan P., Liu A., Chen Y., Wang J., Ruan S., Chen H., Ding J. (2015). Passively Mode-Locked Fiber Laser by a Cell-Type WS_2_ Nanosheets Saturable Absorber. Sci. Rep..

[163] Pogna E.A.A., Marsili M., De Fazio D., Dal Conte S., Manzoni C., Sangalli D., Yoon D., Lombardo A., Ferrari A.C., Marini A. (2016). Photo-Induced Bandgap Renormalization Governs the Ultrafast Response of Single-Layer MoS_2_. ACS Nano.

[164] Yan R., Simpson J.R., Bertolazzi S., Brivio J., Watson M., Wu X., Kis A., Luo T., Hight Walker A.R., Xing H.G. (2014). Thermal Conductivity of Monolayer Molybdenum Disulfide Obtained from Temperature-Dependent Raman Spectroscopy. ACS Nano.

[165] Wang R., Wang T., Zobeiri H., Yuan P., Deng C., Yue Y., Xu S., Wang X. (2018). Measurement of the Thermal Conductivities of Suspended MoS_2_ and MoSe_2_ by Nanosecond ET-Raman without Temperature Calibration and Laser Absorption Evaluation. Nanoscale.

[166] Liu H., Nishide D., Tanaka T., Kataura H. (2011). Large-Scale Single-Chirality Separation of Single-Wall Carbon Nanotubes by Simple Gel Chromatography. Nat. Commun..

[167] Wu K., Zhang X., Wang J., Li X., Chen J. (2015). WS_2_ as a Saturable Absorber for Ultrafast Photonics Applications of Mode-Locked and Q-Switched Lasers. Opt. Express.

[168] Kadir N., Ismail E.I., Latiff A.A., Ahmad H., Arof H., Harun S.W. (2017). Transition Metal Dichalcogenides (WS_2_ and MoS_2_) Saturable Absorbers for Mode-Locked Erbium-Doped Fiber Lasers. Chin. Phys. Lett..

[169] Lu L., Tang X., Cao R., Wu L.M., Li Z.J., Jing G.H., Dong B.Q., Lu S.B., Li Y., Xiang Y.J. (2017). Broadband Nonlinear Optical Response in Few-Layer Antimonene and Antimonene Quantum Dots: A Promising Optical Kerr Media with Enhanced Stability. Adv. Opt. Mater..

[170] Bussolotti F., Chai J., Yang M., Kawai H., Zhang Z., Wang S., Wong S.L., Manzano C., Huang Y., Chi D. (2018). Electronic properties of atomically thin MoS_2_ layers grown by physical vapour deposition: Band structure and energy level alignment at layer/substrate interfaces. RSC Adv..

[171] Nicolosi V., Chhowalla M., Kanatzidis M.G., Strano M.S., Coleman J.N. (2013). Liquid exfoliation of layered materials. Science.

[172] Kauranen M., Zayats A.V. (2012). Nonlinear Plasmonics. Nat. Photonics.

[173] Feng T., Li X., Guo P., Zhang Y., Liu J., Zhang H. (2020). MXene: Two dimensional inorganic compounds, for generation of bound state soliton pulses in nonlinear optical system. Nanophotonics.

[174] Dhasmana N., Fadil D., Kaul A.B., Thomas J. (2016). Investigation of nonlinear optical properties of exfoliated MoS_2_ using Photoacoustic Z-scan. MRS Adv..

[175] Nie Z.G., Long R., Sun L.F., Huang C.C., Zhang J., Xiong Q., Hewak D.W., Shen Z., Prezhdo O.V., Loh Z.H. (2014). Ultrafast Carrier Thermalization and Cooling Dynamics in Few-Layer MoS_2_. ACS Nano.

[176] Liu X., Gao Q., Zheng Y., Mao D., Zhao J. (2020). Recent Progress of Pulsed Fiber Lasers Based on Transition-Metal Dichalcogenides and Black Phosphorus Saturable Absorbers. Nanophotonics.

[177] Grelu P., Akhmediev N. (2012). Dissipative Solitons for Mode-Locked Lasers. Nat. Photonics.

[178] Chhowalla M., Shin H.S., Eda G., Li L.J., Loh K.P., Zhang H. (2013). The Chemistry of Two-Dimensional Layered Transition Metal Dichalcogenide Nanosheets. Nat. Chem..

[179] Guo B., Guo X., Zhou R., Ren Z., Chen Q., Xu R., Luo W. (2023). Multi-Pulse Bound Soliton Fiber Laser Based on MoTe_2_ Saturable Absorber. Nanomaterials.

[180] Krupa K., Nithyanandan K., Andral U., Tchofo-Dinda P., Grelu P. (2017). Real-Time Observation of Internal Motion within Ultrafast Dissipative Optical Soliton Molecules. Phys. Rev. Lett..

[181] Backes C., Higgins T.M., Kelly A., Boland C., Harvey A., Hanlon D., Coleman J.N. (2017). Guidelines for Exfoliation, Characterization and Processing of Layered Materials Produced by Liquid Exfoliation. Chem. Mater..

[182] Rathi S., Lee I., Lim D., Wang J., Ochiai Y., Aoki N., Watanabe K., Taniguchi T., Lee G.-H., Yu Y.-J. (2015). Tunable Electrical and Optical Characteristics in Monolayer Graphene and Few-Layer MoS_2_ Heterostructure Devices. Nano Lett..

[183] Ma Y., Sun H., Ran B., Zhang S., Zhang H., Tittel F.K., Lv Z. (2020). Passively Q-Switched Tm:YAlO_3_ Laser Based on WS_2_/MoS_2_ Two-Dimensional Nanosheets at 2 μm. Opt. Laser Technol..

[184] Chen H., Corboliou V., Solntsev A.S., Choi D.-Y., AVincenti M., de Ceglia D., de Angelis C., Lu Y., Neshev D.N. (2017). Enhanced second-harmonic generation from two-dimensional MoSe_2_ on a silicon waveguide. Light Sci. Appl..

[185] Zhang M., Howe R.C.T., Woodward R.I., Kelleher E.J.R., Torrisi F., Hu G.H., Popov S.V., Taylor J.R., Hasan T. (2014). Solution processed MoS_2_-PVA composite for sub-bandgap mode-locking of a wideband tunable ultrafast Er: Fiber laser. Nano Res..

[186] Wu K., Chen B., Zhang X., Zhang S., Guo C., Li C., Xiao P., Wang J., Zhou L., Zou W. (2018). High-Performance Mode-Locked and Q-Switched Fiber Lasers Based on Novel 2D Materials of Topological Insulators, Transition Metal Dichalcogenides and Black Phosphorus: Review and Perspective (invited). Opt. Commun..

[187] Kumral B., Barri N., Demingos P.G., Adabasi G., Grishko A., Wang G., Kawase J., Onodera M., Machida T., Baykara M.Z. (2025). Mechanically Reliable and Electronically Uniform Monolayer MoS_2_ by Passivation and Defect Healing. Nat. Commun..

[188] Liu P., Pei Q.-X., Zhang Y.-W. (2023). Low-Cycle Fatigue Failure of MoS_2_ Monolayer. Extrem. Mech. Lett..

[189] Pecunia V., Anthopoulos T.D., Armin A., Bouthinon B., Caironi M., Castellanos-Gomez A., Chen Y., Cho K., Clegg C., Fang X. (2025). Guidelines for Accurate Evaluation of Photodetectors Based on Emerging Semiconductor Technologies. Nat. Photonics.

[190] Xie J., Liu T., Liu X., Wang F., Liu W. (2025). Research Progress of Passively Mode-Locked Fiber Lasers Based on Saturable Absorbers. Nanomaterials.

[191] Cun H., Macha M., Kim H., Liu K., Zhao Y., LaGrange T., Kis A., Radenovic A. (2019). Wafer-scale MOCVD growth of monolayer MoS_2_ on sapphire and SiO_2_. Nano Res..

[192] Oh H.M., Han G.H., Kim H., Bae J.J., Jeong M.S., Lee Y.H. (2016). Photochemical Reaction in Monolayer MoS_2_ via Correlated Photoluminescence, Raman Spectroscopy, and Atomic Force Microscopy. ACS Nano.

[193] Lim J., Heo S.J., Jung M., Kim T., Byeon J., Park H.J., Jang J.E., Hong J., Moon J., Pak S. (2024). Highly Sustainable h-BN Encapsulated MoS_2_ Hydrogen Evolution Catalysts. Small.

[194] Bensoussan A., Suhir E., Henderson P., Zahir M. (2015). A unified multiple stress reliability model for microelectronic devices—Application to 1.55 μm DFB laser diode module for space validation. Microelectron. Reliab..

